# A CRISPR Cas9 high-throughput genome editing toolkit for kinetoplastids

**DOI:** 10.1098/rsos.170095

**Published:** 2017-05-03

**Authors:** Tom Beneke, Ross Madden, Laura Makin, Jessica Valli, Jack Sunter, Eva Gluenz

**Affiliations:** Sir William Dunn School of Pathology, University of Oxford, Oxford, UK

**Keywords:** *Leishmania*, *Trypanosoma*, CRISPR, genome editing, T7 RNA polymerase

## Abstract

Clustered regularly interspaced short palindromic repeats (CRISPR), CRISPR-associated gene 9 (Cas9) genome editing is set to revolutionize genetic manipulation of pathogens, including kinetoplastids. CRISPR technology provides the opportunity to develop scalable methods for high-throughput production of mutant phenotypes. Here, we report development of a CRISPR-Cas9 toolkit that allows rapid tagging and gene knockout in diverse kinetoplastid species without requiring the user to perform any DNA cloning. We developed a new protocol for single-guide RNA (sgRNA) delivery using PCR-generated DNA templates which are transcribed *in vivo* by T7 RNA polymerase and an online resource (LeishGEdit.net) for automated primer design. We produced a set of plasmids that allows easy and scalable generation of DNA constructs for transfections in just a few hours. We show how these tools allow knock-in of fluorescent protein tags, modified biotin ligase BirA*, luciferase, HaloTag and small epitope tags, which can be fused to proteins at the N- or C-terminus, for functional studies of proteins and localization screening. These tools enabled generation of null mutants in a single round of transfection in promastigote form *Leishmania major*, *Leishmania mexicana* and bloodstream form *Trypanosoma brucei*; deleted genes were undetectable in non-clonal populations, enabling for the first time rapid and large-scale knockout screens.

## 1. Introduction

Kinetoplastid parasites, including the human pathogenic *Trypanosoma brucei*, *Trypanosoma cruzi* and *Leishmania* spp., pose a huge global burden on health and economic development; thus the need for better diagnostic tools and medicines for these neglected diseases remains urgent. The ability to generate targeted mutations in the laboratory to study mutant phenotypes has been key to the dissection of basic parasite biology, pathogenicity mechanisms and drug resistance. These studies were facilitated by powerful forward and reverse genetics methods [1] and whole-genome sequence information.

For *T. brucei*, important new insights into its basic biology [2–4] and drug-resistance mechanisms [5] emerged from genome-scale loss-of-function screens using RNA interference (RNAi) libraries. *Trypanosoma cruzi* and most *Leishmania* spp. lack essential components of the RNAi machinery [6] and generation of loss-of-function mutants remains, at best, a time-consuming endeavour and largely impossible for many multicopy gene families. Plasticity in chromosome copy numbers [7,8] further complicates targeted mutations of many potentially important genes.

Big ‘-omics’ datasets call for quick methods to interrogate large cohorts of genes to screen, for example, proteomics datasets for subcellular localization of proteins or to identify interaction partners within larger complexes. In *T. brucei*, homologous recombination (HR) can be achieved with targeting sequences short enough for incorporation into oligonucleotide primers for PCR-amplification of gene deletion and tagging cassettes [9–12]. This allowed for development of powerful high-throughput gene-tagging methods [12–14]. *Leishmania* require longer (more than 300 nt) homology arms for efficient and correct integration of donor DNA [12]; targeting cassettes thus have to be assembled in a plasmid by DNA cloning or with a two-step fusion-PCR method, both more labour intensive and less scalable than a one-step PCR protocol.

Genome editing by clustered regularly interspaced short palindromic repeats (CRISPR), CRISPR-associated gene 9 (Cas9) is a transformative new technology [15,16] for precise cleavage of double-stranded DNA by a Cas9 nuclease with sequence-specificity determined by a single-guide RNA (sgRNA). The subsequent repair of the double-strand break provides opportunities for disruption or precise modifications of the target locus. For apicomplexan parasites, CRISPR methods have been used in *Plasmodium* spp. [17,18], *Cryptosporidium* [19] and enabled a genome-wide loss-of-function screen in *Toxoplasma gondii* that identified new genes important in infection of human fibroblasts [20]. The first reports of CRISPR-Cas9 gene editing in kinetoplastids, for *T. cruzi* [16,21,22], *Leishmania donovani* [23] and *Leishmania major* [24], all used similar strategies for expressing Cas9 from episomal plasmids. The target-specific sgRNAs in these studies were delivered either by transfection of *in vitro* transcribed sgRNA [21] or transfection of episomal plasmids for *in vivo* transcription of sgRNAs from RNA Pol I [22,23] or RNA Pol III [24] promoters. Transfection of Cas9 and sgRNA-expressing cells with a donor DNA for homology directed repair produced precise modifications of the target locus [23], allowing for generation of null mutants for single and multicopy genes (e.g. PFR2 in *L. major* [24] and *T. cruzi* [22]) in a single round of transfection and tagging of *T. cruzi* genes at the endogenous locus [25]. Without the addition of donor DNA, Cas9-induced double-strand breaks were repaired by a mechanism of microhomology-mediated end joining (MMEJ), resulting in small deletions at the target site [21,23]. Hence, CRISPR technology enables simultaneous targeting of genes in multicopy families [21,26] or on supernumerary chromosomes, which is not possible with conventional methods.

Our aim was to exploit the efficiency of CRISPR-Cas9-mediated genome modification by integration of donor DNA to develop a simple method for rapid and scalable production of mutant *Leishmania* and other kinetoplastids. We produced *Leishmania mexicana*, *L. major* and *T. brucei* cell lines stably expressing Cas9. Stably expressed T7 RNA polymerase (T7 RNAP) was used to drive sgRNA transcription *in vivo* from DNA templates generated by a single PCR reaction. A set of plasmids serves as PCR templates for rapid production of a variety of donor DNAs without the need for any DNA cloning. We show examples of gene deletion phenotypes affecting flagellar motility, lipophosphoglycan (LPG) production and variant surface glycoprotein (VSG) release, which were obtained in a single round of transfection and observed in non-clonal populations as early as one week after transfection. These tools will facilitate rapid large-scale screening of mutant phenotypes in *Leishmania* and other kinetoplastids.

## 2. Results and discussion

### 2.1. Constitutive expression of Cas9 in *Leishmania mexicana*

**Figure 1.**
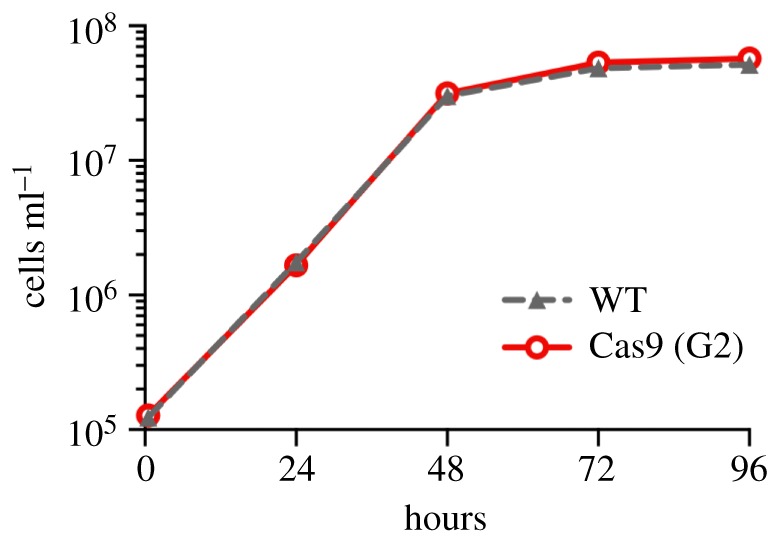
Constitutive expression of Cas9 in *L. mexicana.* Growth curves of *L. mexicana* wild-type cells (WT) and *L. mex* Cas9 clone G2.

To enable CRISPR-Cas9 gene editing, we first engineered *L. mexicana* promastigote forms to express Cas9. We transfected *L. mexicana* with an expression plasmid (pRM006) carrying the humanized *Streptococcus pyogenes* Cas9 nuclease gene (*hSpCas9* [27]) with a nuclear localization signal and three copies of the FLAG epitope tag at the N-terminus, linked to a hygromycin resistance gene. Western blotting confirmed expression of Cas9 in the resulting hygromycin-resistant cells (named *L. mex* Cas9) (electronic supplementary material, figure S1*a*). We found no evidence for Cas9 toxicity: *L. mex* Cas9 promastigote forms grew at the same rate as wild-type cells in log-phase cultures, with doubling times of 6.5 h, and reached stationary phase at the same time and density as the wild-type (figure 1). Their morphology appeared normal by microscopy, and asynchronously growing populations showed the same cell size distribution as the wild-type (electronic supplementary material, figure S1*b*). Constitutive expression of Cas9 thus appeared to be well tolerated by *L. mexicana* promastigotes.

### 2.2. Efficient knock-in of donor DNA with less than 30 nt homology flanks

**Figure 2.**
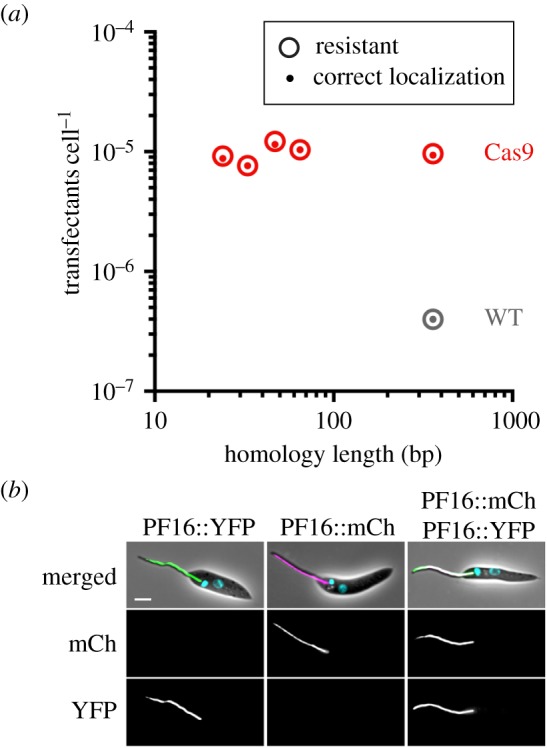
Short HF allow efficient integration of donor DNA. (*a*) *Leishmania mexicana* wild-type cells (WT; grey) and *L. mex* Cas9 clone G2 (red) were transfected with PF16::YFP tagging cassettes containing different length HF (24, 33, 47, 65 and 350 nt). The plot shows the number of transfectants recovered per transfected cell. Large open circles denote blasticidin-resistant cells; small-filled circles denote blasticidin-resistant cells with green fluorescent flagella. For wild-type cells, homology lengths of 24–65 nt yielded no drug-resistant cells. Each data point represents the mean number of transfectants from three independent transfections. (*b*) Micrographs showing PF16::YFP and PF16::mCherry (mCh) expression in *L. mex* Cas9 cells transfected with each tagging construct separately or combined. Merged: phase contrast image overlaid with fluorescence channels showing YFP (green), mCh (magenta) and Hoechst-stained DNA (cyan). Scale bar 5 µm.

We previously showed that integration of DNA fragments by HR in *Leishmania* required homology flanks (HF; sequences at the ends of the donor DNA cassette that are identical to the target locus) of at least 100 nt [12]. Furthermore, there was a linear relationship between success rate (number of correct integration events per transfected cell) and HF length. HF of more than 300 nt were required to achieve useful success rates of at least one correct transfectant per million transfected cells [12]. Since HF of more than 100 nt cannot practically be incorporated into long oligonucleotide primers, this precluded any attempts to generate donor DNA cassettes in a one-step PCR reaction. Previous CRISPR-Cas9 gene editing studies in *Leishmania* spp. reported integration of donor DNA with either very short HF (25 nt; [23,26]) or very long HF (1000 nt [24]) but the effect of HF length on recombination efficiency remained unclear. To address this, we determined the relationship between HF length and rate of correct donor DNA integration in *L. mex* Cas9 promastigotes. We targeted the single copy *PF16* gene (LmxM.20.1400; encoding a central pair protein of the axoneme) with a series of donor DNA cassettes for in-frame tagging with a yellow fluorescent protein gene (*YFP*) linked to a blasticidin-resistance gene with HF ranging from 24–360 nt. *L. mex* Cas9 was then co-transfected with these donor DNA fragments and *in vitro* transcribed sgRNA, targeting a position immediately downstream of the *PF16* CDS. As a control, the same donor DNA fragments were transfected into *L. mexicana* wild-type cells. Following blasticidin selection, the number of drug-resistant cells obtained from each transfection was counted and correct integration of the tagging cassette assessed by observation of a YFP signal in the axoneme (figure 2*a*). Strikingly, in *L. mex* Cas9 cells the frequency of correct integration was independent of HF length; HF as short as 24 nt resulted in correct and efficient tagging of *PF16*. In wild-type cells, HF shorter than 100 nt did not produce any transfectants, consistent with previous results [12], and with 360 nt HF, *L. mex* Cas9 cells yielded more than 10-fold more transfectants than wild-type cells. These results demonstrate that CRISPR-Cas9 greatly enhances the efficiency of donor DNA integration in *Leishmania*. Whether this is mediated by HR or MMEJ was not further investigated here but it is interesting to note that MMEJ was recently reported to play a dominant role in the integration of donor DNA with short HF in *L. donovani* [26].

To test whether CRISPR-Cas9 allowed for targeting of two *PF16* alleles simultaneously with different tags, we generated two DNA cassettes with 30 nt HF for tagging with *YFP*, linked to a blasticidin-resistance gene, or *mCherry*, linked to a neomycin-resistance gene. Co-transfection of the two cassettes with *in vitro* transcribed sgRNA and simultaneous selection with two drugs yielded cells with doubly fluorescent flagella (figure 2*b*) at a rate of 1 × 10^−6^ per transfected cell. These doubly tagged cells remained motile, indicating that expression of two tagged PF16 alleles was compatible with normal flagellar function.

### 2.3. *In vivo* transcription of PCR-amplified sgRNA templates by T7 RNAP

While *in vitro* transcription is a quick method for sgRNA production, we saw an opportunity to simplify the process of sgRNA delivery further by transfecting the double-stranded DNA template used for *in vitro* sgRNA transcription with T7 RNA polymerase (T7 RNAP) [28] directly into cells expressing T7 RNAP. To test this, we inserted a *T7 RNAP* gene [29] into *L. mex* Cas9 and confirmed expression of Cas9 and T7 RNAP in the resulting *L. mex* Cas9 T7 cell line by Western blot (electronic supplementary material, figure S2). These promastigotes appeared morphologically normal and their growth rate was similar to the wild-type and *L. mex* Cas9 cell line (electronic supplementary material, figure S1*c,d*).

**Figure 3.**
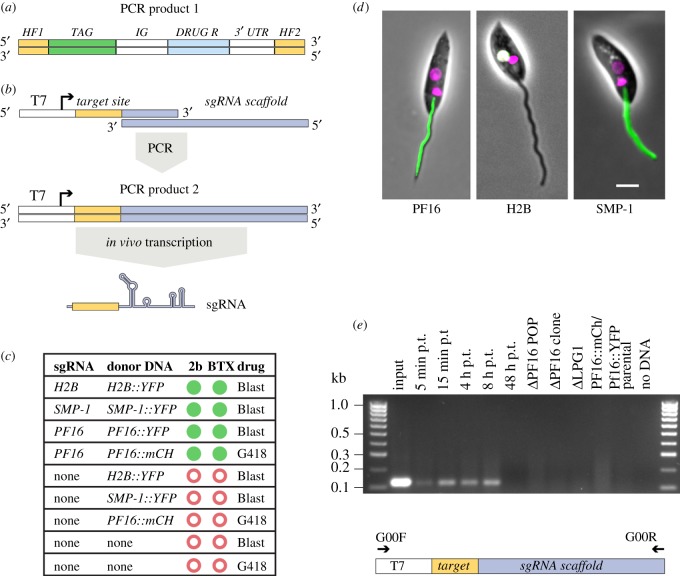
Co-transfection of two PCR amplicons allowed precise insertion of marker genes. (*a*) PCR-amplified donor DNA containing 30 nt HF specific to the target locus, a fluorescent protein tag and a drug-selectable marker gene. (*b*) Strategy for sgRNA delivery: the sgRNA template is produced by PCR using an oligo encoding the T7 promoter, 20 nt defining the target-site and a sequence complementary to the 3′-end of the second oligo, comprising the sgRNA scaffold [28]. The resulting PCR product is transfected into cells for T7 RNAP-driven transcription of the sgRNA. (*c*) Summary of outcome of transfections with different combinations of sgRNA templates and donor DNAs and electroporation protocols. Green filled circles denote drug-resistant cells showing the expected fluorescent signal; red open circles indicate failure to produce any drug-resistant transfectants. (*d*) Micrographs showing correct flagellar localization of PF16::YFP, nuclear localization of H2B::YFP (the white colour indicates co-localization of YFP and Hoechst) and flagellar membrane localization of SMP-1::YFP in cells that were co-transfected with the donor DNA and corresponding sgRNA template. Phase contrast image merged with mCh or YFP fluorescence channels and Hoechst-stained DNA (magenta). Scale bar 5 µm. (*e*) PCR-detection of the sgRNA template. Top, agarose gel showing the results of a diagnostic PCR to test for the presence of the sgRNA template. Template DNAs were as follows. Input: 1 µl of *PF16* sgRNA PCR used for transfection; 5 min-48 h *post* transfection (p.t.): genomic DNA from cells at different time points post transfection with *PF16* sgRNA; Δ*PF16*, Δ*LPG1*, PF16::mCh / PF16::YFP: genomic DNA from drug-resistant cell lines reported in this study; parental: genomic DNA from the parental cell line *L. mex* Cas9 T7. Bottom, diagram showing the sgRNA template and the primers used for PCR detection.

We tested the system by tagging three genes: *PF16*, small myristoylated protein 1 (*SMP-1,* LmxM.20.1310) or histone H2B (LmxM.28.0210). *L. mex* Cas9 T7 cells were co-transfected with donor DNA with 30 nt HF (figure 3*a*) and an sgRNA template for the corresponding gene (figure 3*b*). As controls, cells were transfected with donor DNA alone or without any DNA. We performed these transfections in duplicate to compare the suitability of two transfection platforms, the BTX system and the Amaxa Nucleofector 2b, which is known to enhance transfection efficiency in kinetoplastids [30]. All cells co-transfected with donor DNA and sgRNA template yielded drug-resistant cell lines (figure 3*c*) that showed the expected fluorescence signals in the axoneme (PF16), nucleus (histone H2B) or flagellar membrane (SMP-1) (figure 3*d*). The control transfections yielded no drug-resistant cells. These results indicate that functional sgRNA was transcribed from the linear DNA fragments. In *Plasmodium falciparum*, T7 RNAP has previously been used to transcribe sgRNAs *in vivo* from a circular plasmid whose construction involved production of a target gene-specific sgRNA cassette followed by Gibson assembly [31]. Our simplified protocol for delivery of sgRNA only requires transfection of a PCR product. To test how rapidly the sgRNA templates were lost from a transfected population, DNA isolated at different time points post transfection was analysed by PCR. This showed that sgRNA template DNA remained present up to 8 h post transfection but was undetectable after 48 h (figure 3*e*), indicating loss of the sgRNA template. Our results demonstrate that co-transfection of two PCR products is sufficient for precise modification of a gene locus in *L. mex* Cas9 T7 cells, providing a rapid and scalable new method for gene tagging in *Leishmania*.

### 2.4. pT and pPLOT plasmids enable rapid PCR-generation of donor DNA

**Figure 4.**
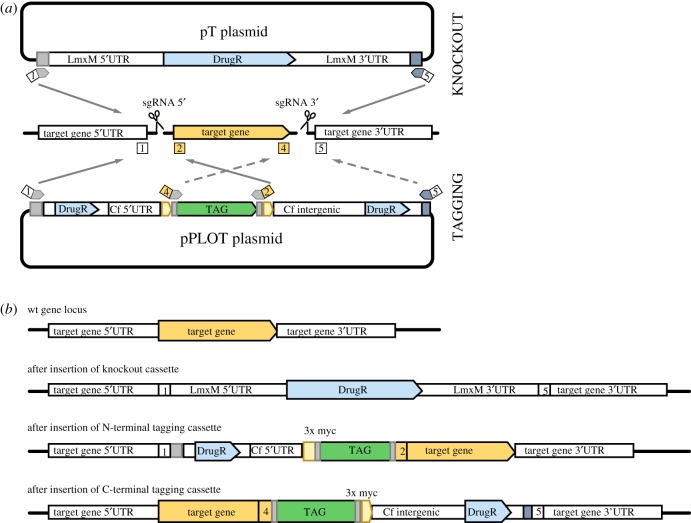
A modular system for PCR-amplification of targeting fragments. (*a*) Strategy for using pT and pPLOT to generate donor DNA for repair of Cas9-induced double-strand breaks allowing precise modification of a target locus. To delete a target gene, two sgRNAs direct cuts to sites immediately upstream (5′) and downstream (3′) of the target gene. Repair cassettes with drug-selectable marker genes (DrugR) and 30 nt HF specific to the target locus are PCR-amplified from pT plasmids with primers 1 and 5. The same primer pair can be used to amplify cassettes with different drug-resistance genes. To tag a target gene, one sgRNA directs a cut immediately upstream or downstream of the target gene, for fusing tags to the N- or C- terminus of a protein, respectively. A repair cassette with 30 nt HF specific to the target locus, the desired tag and a drug-selectable marker gene are PCR-amplified from a pPLOT plasmid. Primer pair 1 and 2 is used for N-terminal tagging (indicated by grey arrows), 4 and 5 for C-terminal tagging (dashed arrows). The same primer pairs can be used to amplify a range of different tagging cassettes. Primers 3 and 6 (not shown) are used to amplify the 5′- and 3′-sgRNA templates. (*b*) Diagrams showing the target gene locus before and after insertion of repair cassettes.

To streamline production of donor DNA for tagging and knockouts (KO), we developed a modular plasmid system where each plasmid serves as a template for PCR-amplification of a drug-selectable repair cassette (figure 4 and table 1; electronic supplementary material, file S1, figure S3, tables S1, S2 and file 5). The pPLOT plasmids for amplification of knock-in cassettes are based on the pPOT plasmid developed for tagging *T. brucei* proteins [12] and allow fusion to the 5′- or 3′-end of a target gene in its endogenous locus, to generate fusion proteins with an N- or C-terminal tag, respectively. Experimental validation of long gene lists from genome analysis, transcriptomics or proteomics studies has to date been very time-consuming. Hence, one major application of pPLOT will be tagging of genes to produce fluorescent fusion proteins for rapid screening of subcellular localization in *Leishmania* spp. Other tags encompassed in the pPLOT plasmid system include small epitope tags for localization studies or immuno-precipitation, Luciferase, the modified biotin ligase BirA* [37] for proximity-dependent biotin labelling and the HaloTag [35] for a variety of studies including imaging, protein purification and identification of interaction partners. pPLOT plasmids are modular to allow for easy expansion of the repertoire by exchanging an existing tag with any sequence of interest and were also designed with all the tags fused to three myc epitopes to simplify detection of fusion proteins on Western blots (electronic supplementary material, figure S4).

**Table 1.** pPLOT plasmids. (Available combinations of fusion tags and resistance genes are indicated by a cross. All tags are fused to a myc epitope. Sequences are in the electronic supplementary material, file S5.)

		resistance			
tag	refs	blasticidin	puromycin	neomycin	phleomycin
mNeonGreen	[32]		×	×	
mCherry	[33]		×		×
10 × TY	[34]		×		
TY::HaloTag::TY	[35]		×		×
NanoLuc luciferase	[36]		×		×
BirA*	[37]		×		

The pT plasmids serve as templates for PCR-amplification of KO cassettes with different drug-selectable markers (Neomycin-kanamycin phosphotransferase, Blasticidin-S deaminase and Puromycin *N*-acetyltransferase). Flanking the drug-resistance genes are *L. mexicana* (LmxM) 5′- and 3′-untranslated region (UTR) sequences, which were selected based on length (maximal 850 nt) and transcript abundance (100–300 fragments per kilobase of transcript per million mapped reads, with similar expression levels in promastigotes and amastigotes [38]) to promote robust expression of the resistance gene independent of target locus and life cycle stage. The UTRs in each plasmid are different (see plasmid maps in the electronic supplementary material, file S1, figure S3 and file 5) to minimize the risk of recombination between KO constructs. The primer binding sites used to amplify the donor DNA are the same for all pT and pPLOT plasmids and compatible with pPOTv4 plasmids [12,13] such that one primer set can be used to amplify any KO or tagging cassette for a specific target gene. This means that a set of just six gene-specific primers (figure 4) and one common sgRNA scaffold primer are sufficient to produce a variety of N- and C-terminally tagged fusion proteins and a null mutant. To facilitate the use of this versatile and economical system for high-throughput tagging and gene deletion, we designed the required primer sequences for the whole genome of several kinetoplastid species (see methods and the electronic supplementary material, figure S5) and developed a simple online tool for downloading the primer sequences at http://leishgedit.net/.

### 2.5. One-step deletion of *Leishmania mexicana PF16* produces immotile flagella

**Figure 5.**
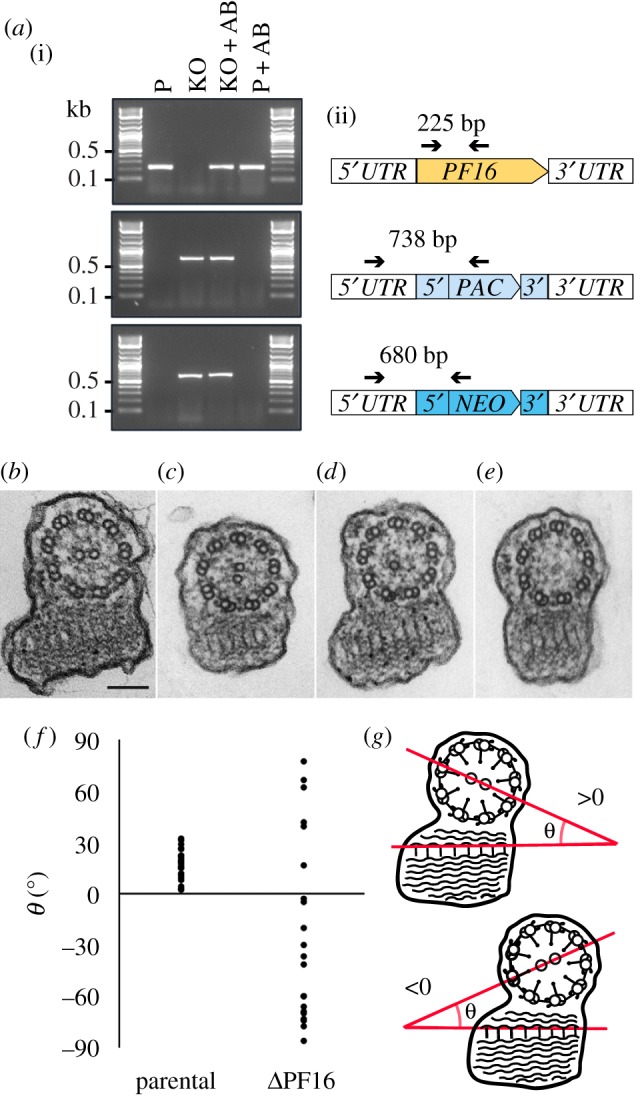
Knockout of *PF16.* (*a*) PCR analysis of the Δ*PF16* cell line. (i) PCR products visualized on agarose gel. P, parental cell line *L. mex* Cas9 T7; KO, Δ*PF16* population; AB, cells expressing an ectopic copy of *PF16*. (ii) Diagram showing the *PF16* locus and PCR primers (arrows) used to test for presence of the *PF16* CDS or the correct integration of the drug-resistance genes (blue boxes). (*b*) Transmission electron microscopy cross section showing the 9 + 2 microtubule arrangement in flagellar axonemes of the parental cell line, scale bar 100 nm. (*c–e*) Axonemes of Δ*PF16* cells with a 9 + 2, 9 + 1 or 9 + 0 microtubule arrangement. (*f*) Measurements of the angle between the plane though the CP and the PFR; parental *N *= 23, Δ*PF16* *N* = 21. (*g*) Cartoon illustrating how angles shown in (*f*) were measured.

Some kinetoplastids are diploid organisms but some species and strains are aneuploid, with chromosome copy number variations. Generation of a null mutant therefore requires excision of at least two copies of a gene. To test whether our tools enabled generation of null mutants in a single round of transfection, we targeted the *PF16* locus again, expecting null mutants to show impaired motility. PF16 is a conserved armadillo repeat protein of motile cilia and flagella, first discovered in mutagenesis screens of *Chlamydomonas reinhardtii* [39,40]. PF16 is attached to the C1 microtubule of the central pair [40] and required for C1 stability and motility [39]. To produce *PF16* KO, *L. mexicana* Cas9 T7 was transfected with two sgRNA templates to generate a double-strand break upstream and downstream of the *PF16* CDS, and with two repair cassettes generated from pT plasmids. All possible combinations pTBlast/pTNeo, pTBlast/pTPuro and pTNeo/pTPuro were used and yielded similar results. The following analysis refers to KO generated with the combination pTPuro/pTNeo. We employed dual drug selection to eliminate any heterozygous cells that still harboured a copy of *PF16*. Microscopic examination of double drug-resistant cell populations 6–7 days after transfection found that these cells were immotile (electronic supplementary material, files S2 and S3). PCR analysis confirmed that the *PF16* CDS was undetectable and both drug-resistance genes were linked to the *PF16* locus in these mutants (figure 5*a*). Sequencing of PCR amplicons spanning the resistance cassette integration sites in the Δ*PF16* population and a Δ*PF16* clone showed that integration of the cassettes was precise, with no detectable insertions or deletions (electronic supplementary material, figure S6) and the sgRNA template had been lost (figure 3*e*). These data suggest that loss of motility was caused by deletion of *PF16*. Transmission electron microscopy of the Δ*PF16* population revealed that instead of the normal 9 + 2 microtubule axoneme (figure 5*b*), many axonemal profiles showed a 9 + 1 or 9 + 0 configuration (figure 5*d,e*), consistent with the role of PF16 in stabilization of central pair (CP) microtubules [39]. The 9 + 2 axonemes that remained in the Δ*PF16* population (figure 5*c*) were unlikely to represent a subpopulation of cells that had retained a copy of *PF16*. Measurements of the angle between the paraflageller rod (PFR) and the plane bisecting the two CP microtubules in Δ*PF16* 9 + 2 axonemes showed great variation in CP orientation, with angles ranging from −90° to +90° (mean −22.3° ± 53.5°). By contrast, in the parental cell line the CP was fixed at a mean angle of 17.2° (±8.5°) (figure 5*f,g*), consistent with earlier reports of a fixed CP orientation in kinetoplastids [41,42]. Our data indicate that the constraints on CP orientation had been lost in the Δ*PF16* mutant, similar to the phenotype observed following RNAi against *PF16* in *T. brucei* [41,43]. To restore *PF16* expression, an ectopic copy of *PF16* was introduced into Δ*PF16* cells. This restored normal motility (electronic supplementary material, file S4), confirming that the observed phenotype was because of the loss of *PF16* and not a consequence of undefined off-target effects of CRISPR-Cas9 genome editing.

### 2.6. One-step deletion of *Leishmania mexicana LPG1*

The same KO strategy was used to delete another gene, *LPG1* (LmxM.25.0010), which encodes a galactofuranosyl transferase required for the biosynthesis of the abundant promastigote cell surface molecule LPG. LPG plays important roles in interactions between promastigotes and the sand fly [44], and protects *Leishmania* from complement lysis and modulates the immunological response to infection (reviewed in [45]). *LPG1* has previously been deleted in *L. major* [46] and *L. mexicana* promastigotes [47] resulting in loss of LPG from the surface and accumulation of a truncated LPG (Glc-P-Man_2_-GlcN-PI), lacking the phosphoglycan chain*.*

**Figure 6.**
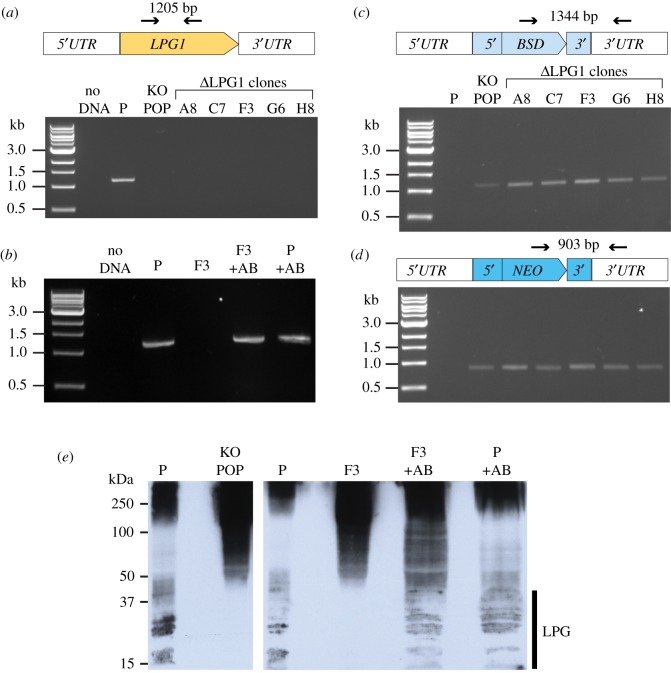
Knockout of *LPG1.* (*a,b*) PCR analysis of the Δ*LPG1* cell line: test for the presence of the *LPG1* CDS; (*c*) test for correct integration of the blasticidin-resistance gene; (*d*) test for correct integration of the neomycin-resistance gene, lanes as in (*c*). Diagrams above the gel pictures show the primers (arrows) used for PCR and size of expected product. (*e*) Western blot of whole-cell lysates probed with LT22. P, parental cell line *L. mex* Cas9 T7; KO POP, Δ*LPG1* population; F3, Δ*LPG1* clonal cell line; +AB, cell lines expressing an ectopic copy of *LPG1*.

Following co-transfection of *L. mex* Cas9 T7 with two *LPG1* sgRNA targeting cassettes and two repair cassettes amplified from pTNeo and pTBlast, genomic DNA from the double drug-resistant population and five clones was examined and confirmed loss of the *LPG1* CDS (figure 6*a*), correct integration of the blasticidin- (figure 6*c*) and neomycin- (figure 6*d*) resistance cassettes and loss of the sgRNA template (figure 3*e*). An ectopic copy of *LPG1* (figure 6*b*) introduced into Δ*LPG1* clone F3 provided an add-back control cell line, Δ*LPG1* [*LPG1*]. To test for the expression of LPG, Western blots of *L. mex* Cas9 T7, Δ*LPG1* and Δ*LPG* [*LPG1*] cell lysates were probed with the monoclonal antibody LT22 which recognizes glucosylated phosphosaccharide repeats of *L. mexicana* LPG [48]. In the parental cell line, LT22 detected the expected pattern of higher molecular weight phosphoglycan compounds and LPG. LPG was undetectable in the uncloned Δ*LPG1* population and clonal Δ*LPG1* cell lines but again present in the addback cell line Δ*LPG* [*LPG1*]*,* at similar levels to the parental cell line (figure 6*e*). The Δ*LPG1* cells showed a stronger signal for the higher molecular weight phosphoglycan compounds than the parental cell line. This is probably a result of the upregulation of proteophoshpoglycans in *L. mexicana LPG1* deletion mutants, first reported by Thomas Ilg [47]. These results indicate successful deletion of *LPG1* and restoration of LPG synthesis in the add-back cell line.

### 2.7. Rapid genome editing in *Leishmania major*

To facilitate adoption of CRISPR-Cas9 editing methods in different kinetoplastid species, we generated a range of plasmids for expression of Cas9 and T7 RNAP (listed in the electronic supplementary material, file S1 and table S3). pTB007, a single-marker plasmid for expression of hSpCas9 and T7RNAP in *Leishmania* spp. was transfected into *L. major* promastigotes and the resulting *L. major* pTB007 cells expressed levels of Cas9 and T7RNAP similar to *L. mex* Cas9 T7, with no detrimental effect on cell growth (electronic supplementary material, figure S2 and S7*a,b*). To test the functionality of the *L. major* pTB007 cell line, the *PF16* (LmjF.20.1400) locus was edited, using PCR-generated sgRNA templates co-transfected with pPLOT- or pT-derived amplicons. pPLOT tagging of PF16 with mNeonGreen (mNG) resulted in fluorescent flagella (electronic supplementary material, figure S7*c*); integration of pTBlast and pTPuro cassettes successfully replaced the *PF16* CDS (electronic supplementary material, figure S7*d*) and motility was severely impaired in the resulting Δ*PF16* population, as seen with the *L. mex* Cas9 T7 Δ*PF16* cells.

### 2.8. Rapid tagging and deletion of GPI-PLC in *Trypanosoma brucei* bloodstream forms

**Figure 7.**
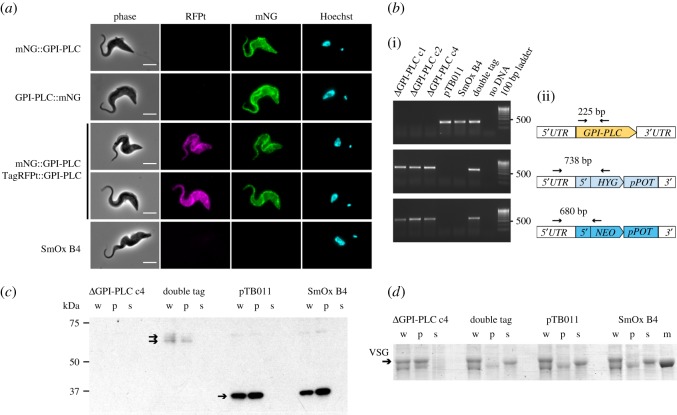
Targeting *GPI-PLC* in *T. brucei* bloodstream forms. (*a*) Localization of tagged GPI-PLC in *T. brucei* SmOx B4 Cas9 cells co-transfected with sgRNA templates and donor DNA(s); one or both alleles of GPI-PLC were tagged with mNeonGreen (mNG) or TagRFP S158 T ([51], TagRFPt), as indicated. SmOx B4 is the parental untagged cell line. Scale bar 5 µm. (*b*) PCR analysis of the following cell lines: three independent Δ*GPI-PLC* clones, doubly tagged cell line mNG::GPI-PLC/TagRFPt::GPI-PLC, SmOx B4 pTB011 and SmOx B4. (i) PCR amplicons visualized on an agarose gel. (ii) diagrams showing the primers (arrows) used for PCR and size of expected product. (*c,d*) Cells were subjected to hypotonic lysis for 20 min, separated into pellet (p) and supernatant (s) fractions and run together with whole-cell lysates (w) on an SDS PAGE gel. (*c*) Western blot probed with anti-GPI-PLC (arrows indicate expected bands: TagRFPt::GPI-PLC, 67.5 kDa; mNG::GPI-PLC 66.5 kDa; GPI-PLC, 40 kDa); (*d*) Coomassie stained gel (arrow indicates VSG; m, 50 kDa marker).

To enable the use of CRISPR tools in *T. brucei*, we introduced hSpCas9 into the *T. brucei* T7RNAP-expressing cell lines SmOx B4 (bloodstream form) and SmOx P9 (procyclic form) (electronic supplementary material, figures S2 and S8*a*; [49]). Cas9 expression had no effect on growth rate (electronic supplementary material, figure S8*b,c*). To test whether our tools allowed rapid gene editing in *T. brucei* SmOx B4 Cas9, we targeted glycosylphosphatidylinositol-specific phospholipase C (GPI-PLC, Tb927.2.6000), a virulence factor required for the release of the GPI-anchored VSG from dying cells [50]. In cells that had been co-transfected with a PCR-generated sgRNA template and one or two fluorescent protein tagging cassettes with 30 nt HF amplified from the pPOTv4 template ([12], electronic supplementary material, figure S8*d*), tagged GPI-PLC localized to both the flagellum and cell body membrane with enrichment on the former (figure 7*a*). This was similar to the localization of YFP-tagged GPI-PLC in an earlier report [52]. Transfections with the tagging constructs alone yielded no drug-resistant cells, indicating that 30 nt HF were too short to generate recombinants in the absence of a sgRNA template. Co-transfection of two sgRNA templates and two drug-resistance cassettes (amplified from pPOT, electronic supplementary material, figure S8*d*) resulted in deletion of *GPI-PLC* in a single step (figure 7*b*). Western blot analysis of the three independent Δ*GPI-PLC* clones confirmed that they did not express any GPI-PLC protein (figure 7*c*). Subjected to a hypotonic lysis assay, Δ*GPI-PLC* cells were unable to release soluble VSG into the medium, consistent with the phenotype of *GPI-PLC* null mutants produced by conventional sequential KO [50]. By contrast, loss of VSG from the pellet fraction and accumulation in the supernatant was observed in the Cas9 expressing parental cell line and SmOx B4 cells and the cell line with two tagged copies of GPI-PLC, indicating these remained functional (figure 7*d*). These results show that in *T. brucei* bloodstream forms, like in *Leishmania*, Cas9 facilitates integration of donor DNA with short HF and enables rapid modification or deletion of two alleles in a single transfection.

## 3. Conclusion

Here, we report a CRISPR-Cas9 toolkit for rapid and precise gene modification by integration of donor DNA, using engineered cell lines and drug selection of mutants. All required sgRNA and donor DNA cassettes are generated by PCR in just a few hours without time-consuming DNA cloning. Remarkably, one week after co-transfection of the relevant PCR products, *PF16* and *LPG1* were undetectable in non-clonal drug-resistant populations. Our simple method for mutant generation allows many cell lines to be produced in parallel; PCRs and transfections can be performed in multi-well plates, enabling rapid and high-throughput screening of knockout phenotypes in *Leishmania* spp. and trypanosomes.

We have shown that these tools and protocols are applicable to different kinetoplastid species, including *T. brucei* bloodstream forms. Our methods provide a platform for comparison of editing efficiencies obtained for different sgRNA target sites or with different promoters for sgRNA transcription, and will help to exploit other CRISPR-related technologies based on a variety of modified forms of the Cas9 nuclease [53] to study the biology of these parasites. Our method can already be adapted for construction of pooled screening libraries through introduction of barcodes into the repair cassettes and should, with further development, enable genome-wide knockout screens. One priority is to refine methods for precise marker-free editing of gene loci, for example, to introduce point mutations without altering the genomic context and to increase the efficiency of mutations by MMEJ. We also plan to develop an inducible system to control the timing of Cas9 cleavage, since the study of genes essential for viability is currently not straightforward with the method described in this paper. CRISPR-Cas9 editing promises to overcome major barriers to the study of gene function in kinetoplastids and will, for the first time to our knowledge, enable large-scale loss-of-function screens in *Leishmania* spp. and *T. cruzi*.

## 4. Material and methods

### 4.1. Cell culture

Promastigote form *L. mexicana* (WHO strain MNYC/BZ/62/M379), *L. major* Friedlin and their genetically modified derivatives were grown at 28°C in M199 medium (Life Technologies) supplemented with 2.2 g l^−1^ NaHCO_3_, 0.005% haemin, 40 mM 4-(2-Hydroxyethyl)piperazine-1-ethanesulfonic acid pH 7.4 and 10% FCS. Relevant selection drugs (Melford Laboratories Ltd.) were added to the medium at the following concentrations: 32 µg ml^−1^ Hygromycin B, 20 µg ml^−1^ Puromycin Dihydrochloride, 5 µg ml^−1^ Blasticidin S Hydrochloride, 40 µg ml^−1^ G-418 Disulfate, 50 µg ml^−1^ Nourseothricin Sulfate and 25 µg ml^−1^ Phleomycin. *Trypanosoma brucei* SmOx B4 [49] was grown in HMI-9 [54] at 37°C in 5% CO_2_, *T. brucei* SmOx P9 in SDM-79 [55] at 28°C. Relevant selection drugs (Melford Laboratories Ltd.) were added to the medium at the following concentrations: 0.2 µg ml^−1^ Puromycin Dihydrochloride, 5 µg ml^−1^ Blasticidin S Hydrochloride, 5 µg ml^−1^ G-418 Disulfate, 5 µg/ml Hygromycin B. Cell culture densities were measured using a CASY model TT cell counter (Roche Diagnostics) with a 60 µm capillary and exclusion of particles with a pseudo diameter below 2.0 µm.

### 4.2. Transfections

Unless otherwise stated, cells were transfected in 1× Tb-BSF buffer [30] using one pulse with program X-001 in the Amaxa Nucleofector IIb (Lonza).

For calculation of recombination rate, 10^7^ *L. mex* Cas9 cells were transfected with 10^5^ molecules of respective DNA fragment per cell and 40 µg *in vitro* transcribed RNA. Cells were recovered in M199 for 8 h, then blasticidin was added and cells were plated on a 96-well plate. The mean rate of resistance from three independent transfections was calculated from the positive wells on plates seeded with 5 × 10^6^ cells and 5 × 10^5^ cells.

For double tagging of *PF16*, 10^7^ *L. mex* Cas9 cells were co-transfected with 3 µg of donor DNA and 30 µg *in vitro* transcribed sgRNA for single tags or with 5 µg of each donor DNA and 100 µg sgRNA for double tagging.

For tests of *in vivo* sgRNA transcription, *L. mex* Cas9 T7 cells were transfected with 4 µg donor DNA and 4 µg sgRNA template DNA. Transfections with the Amaxa Nucleofector IIb used 3 × 10^6^ cells. For transfections with the BTX ECM 830 square wave electroporation system, 10^7^ cells were pulsed three times with 1.5 kV (unipolar, 100 µs, 10s interval) in 0.4 cm gap Gene Pulser electroporation cuvettes (BioRad).

For gene tagging with pPLOT constructs, 3 × 10^6^ cells were transfected with the PCR reactions for the sgRNA and donor DNA (combined volume approx. 50 µl) in a total volume of 250 µl. For gene KOs with pT constructs, 10^7^ cells were transfected with the PCR reactions for the two sgRNAs and two donor DNAs (combined volume approx. 100 µl) in a total volume of 250 µl.

For *T. brucei* gene tagging, PCR products were ethanol precipitated, DNA pellets resuspended in a total volume of 100 µl Tb-BSF buffer and mixed with 10^7^ cells in 100 µl Tb-BSF buffer.

Electroporated cells were immediately transferred into pre-warmed medium and left to recover for 4–16 h before adding selection drugs. Survival of drug-resistant transfectants typically became apparent 5–7 days after transfection.

### 4.3. Construction of Cas9 and T7 RNAP expression plasmids

For expression of Cas9 in *Leishmania*, plasmid pRM006 was constructed: humanized *S. pyogenes* Cas9 from plasmid pX330 (Addgene #42230; [27]) was joined to *Crithidia fasciculata* PGKB 5′-UTR and GSPS 3′-UTR from pLENT V1 [12] and the backbone of pLENT V1, containing a phleomycin-resistance gene, which was subsequently replaced with a hygromycin B phosphotransferase gene. Finally, fragments from the 5′- and 3′-UTR of *L. major* β-tubulin from a constitutive expression plasmid [14] were inserted and the plasmid was linearized with *Pac* I before transfection. Insertion of the T7 RNAP cassette with calmodulin UTRs from pVY087 [29] into pRM006, downstream of Cas9 generated pTB007. For expression of Cas9 in *T. brucei*, hSpCas9 from pX330 was inserted into pPOTv4-Puro-mNG-Puro [12] between the aldolase and PFR2 intergenic sequences, replacing the mNG gene. The fragment containing the puromycin-resistance gene and Cas9 expression cassette was subsequently excised and inserted into a plasmid containing a blasticidin-resistance gene and targeting fragments for the *T. brucei* tubulin locus. The resulting plasmid pTB011 was linearized with *Pac* I before transfection. Plasmid sequences are in the electronic supplementary material, file S5.

### 4.4. Construction of pT and pPLOT plasmids

For the construction of pT plasmids, expression cassettes for the Neomycin-kanamycin phosphotransferase (*NEO*), Blasticidin-S deaminase (*BSD*) or Puromycin *N*-acetyltransferase (*PAC*) gene were made by fusion PCR [12]; primer sequences are detailed in the electronic supplementary material, file S1. In the first reaction, UTRs were amplified from the *L. mexicana* genome, in the second reaction, the UTRs were fused to the drug-resistance genes. The resulting PCR product was joined to the *Swa* I fragment of B6162 [56] containing the ampicillin-resistance gene and high-copy-number pUC origin. Plasmid sequences are in the electronic supplementary material, file S5.

pPOTv4 [12] was used as backbone to generate pPLOT plasmids. First a triple myc epitope tag was amplified by using two overlapping primers. This amplicon (approx. 150 bp) was used in a second PCR as a long primer to amplify the *C. fasciculata* PGKB 5′-UTR or PGKA/B intergenic sequence (IG) from pLENTv1 [12]. Products, containing the 5′-UTR or IG linked to a triple myc tag, were cloned into pPOTv4 blast–blast mNeonGreen to make pPLOT blast–blast mNeonGreen. To generate pPLOT variants with different resistance markers, the drug-resistance backbone, containing both blasticidin-resistance genes, was exchanged with other drug-resistance backbones of pPOTv4 plasmids using *Nhe* I and *Mlu* I restriction sites. To generate pPLOT variants with different tags, mNeonGreen was replaced with BirA*, mCherry, 10×TY, TY::HaloTag::TY and NanoLuc Luciferase using *Hin* dIII and *Bam* HI sites. Plasmid sequences are in the electronic supplementary material, file S5.

### 4.5. Construction of addback plasmids

The *PF16* or *LPG1* CDS were amplified, cloned into the *Eco* RI and *Spe* I sites of pRM005 (a version of pRM006 with a phleomycin-resistance gene; for plasmid sequence, see the electronic supplementary material, file S5) and integrated into the *β-tubulin* locus. Primer sequences are detailed in the electronic supplementary material, file S1. Cells transfected with addback plasmids were incubated both with phleomycin, for selection stable transfectants, and hygromycin, for maintenance of the pRM006 Cas9 cassette, which contained the same targeting sites as pRM005.

### 4.6. PCR-amplification of targeting fragments

For recombination rate measurements, PCR-amplification of donor DNA for *PF16* tagging used the same primers as [12]. The DNA template was pLENT-PF16-YFPTY_Var4.4, derived from pLENT-PF16::eGFP [12] by replacing the *eGFP*-*PGKA-PGKB* UTR cassette with *eYFP* and the IG downstream of LmxM.34.1670 (60S ribosomal protein L26). For tests of *in vivo* sgRNA transcription, *eYFP* cassettes with HF targeting *SMP-1*, *histone 2B* or *PF16* were PCR-amplified from pLENT-PF16-YFPTY_Var4.4. For double tagging of *PF16*, the *mCherry* and *eYFP* cassettes were PCR-amplified from pLENTv1 and v2 [12], respectively.

For amplification of pPLOT and pT cassettes, 30 ng circular pPLOT or pT plasmid, 0.2 mM dNTPs, 2 µM each of gene-specific forward and reverse primers and 1 unit HiFi Polymerase (Roche) were mixed in 1× HiFi reaction buffer with MgCl_2_ (Roche), supplemented with 1.875 mM MgCl_2_ (final MgCl_2_ concentration, 3.375 mM) and 3% (v/v) DMSO, 40 µl total volume. PCR steps were 5 min at 94°C followed by 40 cycles of 30 s at 94°C, 30 s at 65°C, 2 min 15 s at 72°C followed by a final elongation step for 7 min at 72°C. Two microlitres of this reaction was run on a 1% agarose gel to check for the presence of the expected product. The remainder was heat-sterilized at 94°C for 5 min and used for transfection without further purification.

For targeting of GPI-PLC in *T. brucei SmOx B4*, targeting cassettes were amplified from pPOTv4 plasmids [12] using the same PCR conditions as above.

### 4.7. PCR-amplification of sgRNA templates

For amplification of sgRNA templates, 0.2 mM dNTPs, 2 µM each of primer G00 (sgRNA scaffold) and a gene-specific forward primer and 1 unit HiFi Polymerase (Roche) were mixed in 1× HiFi reaction buffer with MgCl_2_ (Roche), 20 µl total volume. PCR steps were 30 s at 98°C followed by 35 cycles of 10 s at 98°C, 30 s at 60°C, 15 s at 72°C. Two microlitres of this reaction was run on a 2% agarose gel to check for the presence of the expected product. The remainder was heat-sterilized at 94°C for 5 min and transfected without further purification. Primer sequences are detailed in the electronic supplementary material, file S1.

### 4.8. Diagnostic PCRs

To screen for loss of the target gene in KO cell lines, genomic DNA was isolated 10–14 days post transfection with the DNeasy Blood & Tissue Kit (Qiagen). One hundred nanograms of isolated DNA was mixed with 0.2 mM dNTPs, 2 µM forward primer and reverse primer, 1 unit HiFi Polymerase (Roche) and 1× HiFi reaction buffer supplemented with MgCl_2_ (Roche), 20 µl total volume. PCR steps were 5 min at 94°C followed by 35 cycles of 30 s at 94°C, 5 s at 60°C, 50 s at 72°C followed by a final elongation step for 7 min at 72°C. Five microlitres of this reaction was run on a 2% agarose gel to check for the presence of the expected product. To test for the presence of the sgRNA template in cells, genomic DNA was isolated from established cell lines or from freshly transfected cultures and analysed by PCR as described above, except that PCR steps were 5 min at 94°C followed by 30 cycles of 20 s at 94°C, 10 s at 65°C, 15 s at 72°C followed by a final elongation step for 7 min at 72°C. Primer sequences are detailed in the electronic supplementary material, file S1.

### 4.9. Sanger sequencing

To sequence construct integration sites, PCR amplicons were run on a 2% agarose gel, the band excised from the gel and purified using the QIAquick Gel Extraction Kit (Qiagen). Sanger sequencing of the amplicon was performed by Source BioScience. Primer sequences are detailed in the electronic supplementary material, file S1.

### 4.10. Automated design of sgRNA and primers

For the design of sgRNA primers, a 45 bp window upstream of the start codon and downstream of the stop codon was searched for a guide RNA sequence. The Eukaryotic Pathogen CRISPR guide RNA/DNA Design Tool (http://grna.ctegd.uga.edu) was used to identify guide RNA sequences, using the default parameters (SpCas9: gRNA length 20; PAM: NGG; off-target PAM: NAG, NGA) and the highest scoring 20 nt guide RNA sequence was selected. If no guide RNA site was found, the window was extended to 75 bp and then to a maximal distance of 105 bp. Primers for amplification of the targeting cassettes used in this study were designed to contain 30 nt of sequence immediately adjacent to the guide RNA search window. To reduce the distance between the Cas9 cut site and the HF, the primer design was subsequently optimized by placing the 30 nt HF sequence immediately adjacent to the guide RNA target sequence. This approach identified primers for 9097 of 9169 annotated genes in *L. mexicana* (using RNA-seq based gene models defined in [38], excluding only 72 genes owing to gaps in the current genome assembly), 8390 of 8400 *L. major* Friedlin genes, 7861 of 8195 *L. donovani* BPK282A1 genes, 11153 of 11567 *T. brucei* TREU 927 genes and 9230 of 11348 *T. cruzi* Dmc28 genes. Primer sequences can be downloaded from http://leishgedit.net/. To assess the specificity of each guide RNA, the identified sequences (23 nt, including the protospacer adjacent motif NGG) were aligned against the respective genome and the number of matches was recorded for each guide RNA (electronic supplementary material, figure S5), confirming that the vast majority of selected sites are unique in the genome.

### 4.11. *In vitro* transcription of sgRNA

sgRNA was generated by *in vitro* transcription of PCR-generated sgRNA templates [28] using the MEGAshortscript T7 Transcription Kit (Thermo Fisher Scientific) following the manufacturer's protocol.

### 4.12. Western blots

Cell lysates were prepared by washing cells once in phosphate buffered saline (PBS) then heating in 1× Laemmli buffer for either 10 min at 60°C (for detection of FLAG, T7 RNAP and myc), 3 min at 100°C (detection of LPG) or 5 min at 100°C (detection of GPI-PLC). Approximately 4 × 10^6^ cell equivalents for *Leishmania* promastigotes and 1 × 10^6^ cell equivalents for *Trypanosoma* parasites were loaded and subjected to electrophoresis on 10% SDS-polyacrylamide gels and transferred to nitrocellulose membranes (GE Healthcare). The membranes were incubated in blocking buffer for 1 h then probed for 1 h with primary antibody, washed in Tris buffered saline with 0.05% (w/v) Tween 20 (TBST), incubated for 1 h with secondary horseradish peroxidase-conjugated antibody, washed in TBST and visualized by addition of the ECL chemiluminescent detection system (PerkinElmer) and exposure to autoradiography film (Carestream). Details of antibodies, dilutions and buffers can be found in the electronic supplementary material, file S1.

### 4.13. Hypotonic cell lysis

Hypotonic cell lysis was performed similarly to [52]. 1 × 10^7^ cells were harvested from a culture at 1 × 10^6^ cells ml^–1^. Cells were washed in 1 ml PBS supplemented with 10 mM glucose and 46 mM sucrose, pH 7.6 (vPBS) and centrifuged again at 800 *g* for 10 min. The cells were resuspended in 100 µl of 0.1 mM Tosyl-l-lysyl-chloromethane hydrochloride (TLCK; Sigma) diluted in water and incubated for 20 min at room temperature. Samples were centrifuged at 14 000*g* for 4 min. Subsequently, supernatant and pellet were separated and 5× Laemmli buffer was added to a final concentration of 1× respectively. Samples were boiled and 1 × 10^6^ cell equivalents analysed by SDS PAGE and Western blotting as above. Gels were stained for 30 min with Coomassie brilliant blue (Sigma).

### 4.14. Fluorescence microscopy

*Leishmania* cells expressing fluorescent fusion proteins were imaged live. Samples were prepared as described in [57]. For *T. brucei* bloodstream forms, cells from 1 ml of culture (1 × 10^6^ cells ml^−1^) were centrifuged at 800*g* for 3 min, washed in 1 ml vPBS, centrifuged again and resuspended in 30 µl vPBS containing 4% (v/v) formaldehyde. Cells were immediately imaged with a 63× NA 1.4 objective lens on a DM5500 B microscope (Leica Microsystems) with a Neo sCMOS camera (Andor Technology) at the ambient temperature of 25–28°C.

### 4.15. Transmission electron microscopy

Cells in mid-log phase were fixed in 2.5% glutaraldehyde by addition of 1/10 volume of 25% glutaraldehyde directly to the culture and incubated at room temperature for 5 min. Fixed cells were post-fixed in osmium tetroxide, stained with uranyl acetate and embedded in Agar 100 epoxy resin as described in [58]. Sections (90 nm) were cut on an Ultracut 7 microtome (Leica), post stained with lead citrate and imaged in a Tecnai 12 transmission electron microscope (Hillsboro, OR, USA) at 120 kV. Images were captured with a Gatan OneView 4 k × 4 k CMOS camera using Gatan Microscopy Suite 3 software (Gatan, USA) and processed in ImageJ.

## Supplementary Material

Supplementary Tables, Figures and Methods

## Supplementary Material

Plasmid sequences
