# Supplementary material for: A CRISPR Cas9 high-throughput genome editing toolkit for kinetoplastids
Source: R Soc Open Sci. 2017 May 3;4(5):170095. doi: 10.1098/rsos.170095 (PMC5451818; doi:10.1098/rsos.170095)
Supplement: Supplementary Tables, Figures and Methods [file rsos170095supp1.pdf]

Supplementary Tables

Table S1. List of pPLOT plasmids

Name	Resistance	5' tagging cassette		3' tagging cassette	
		tags	size (kb)	tags	size (kb)
pPLOT-**mNG** blast-blast	BLAST	$Myc_{(3)}$::mNeonGreen	2.1	mNeonGreen::$Myc_{(3)}$	2.5
pPLOT-**mNG** neo-neo	NEO	$Myc_{(3)}$::mNeonGreen	2.5	mNeonGreen::$Myc_{(3)}$	2.9
pPLOT-**mNG** puro-puro	PURO	$Myc_{(3)}$::mNeonGreen	2.3	mNeonGreen::$Myc_{(3)}$	2.7
pPLOT-**mCherry**-phleo-phleo	BLE	$Myc_{(3)}$::mCherry	2.1	mCherry::$Myc_{(3)}$	2.5
pPLOT-**mCherry**-puro-puro	PURO	$Myc_{(3)}$::mCherry	2.3	mCherry::$Myc_{(3)}$	2.7
pPLOT-**Halo**-phleo-phleo	BLE	$Myc_{(3)}$::Ty::Halo::Ty	2.3	Ty::Halo::Ty::$Myc_{(3)}$	2.7
pPLOT-**Halo**-puro-puro	PURO	$Myc_{(3)}$::Ty::Halo::Ty	2.4	Ty::Halo::Ty::$Myc_{(3)}$	2.8
pPLOT-10**Ty**-puro-puro	PURO	$Myc_{(3)}$::$Ty_{(10)}$	2.0	$Ty_{(10)}$::$Myc_{(3)}$	2.3
pPLOT-**nanoLuc**-puro-puro	PURO	$Myc_{(3)}$::Luciferase	2.1	Luciferase::$Myc_{(3)}$	2.5
pPLOT-**nanoLuc**-phleo-phleo	BLE	$Myc_{(3)}$::Luciferase	1.9	Luciferase::$Myc_{(3)}$	2.3
pPLOT- **BirA***-puro-puro	PURO	$Myc_{(3)}$::BirA*	2.6	BirA*::$Myc_{(3)}$	3.0
pPLOT- **BirA***-phleo-phleo	BLE	$Myc_{(3)}$::BirA*	2.4	BirA*::$Myc_{(3)}$	2.8

Table S2. List of pT plasmids

Name	Resistance	knockout cassette size (kb)
pTBlast	BLAST	1.7
pTNeo	NEO	1.75
pTPuro	PURO	1.8

Table S3. List of expression plasmids for Cas9, T7 RNAP, and addback

Name	Resistance	protein expression	targeting sequences	species
**pRM006**	HYG	hSpCas9	β-tubulin array	*L. major*
**pTB007**	HYG	hSpCas9, T7 RNAP	β-tubulin array	*L. major*
**pTB008**	PHLEO	T7 RNAP	SSU rRNA	*Leishmania* spp.
**pTB011**	PURO BLAST	hSpCas9	β-tubulin array	*T. brucei*
**pTadd**	PHLEO	(experiment-specific)	β-tubulin array	*L. major*

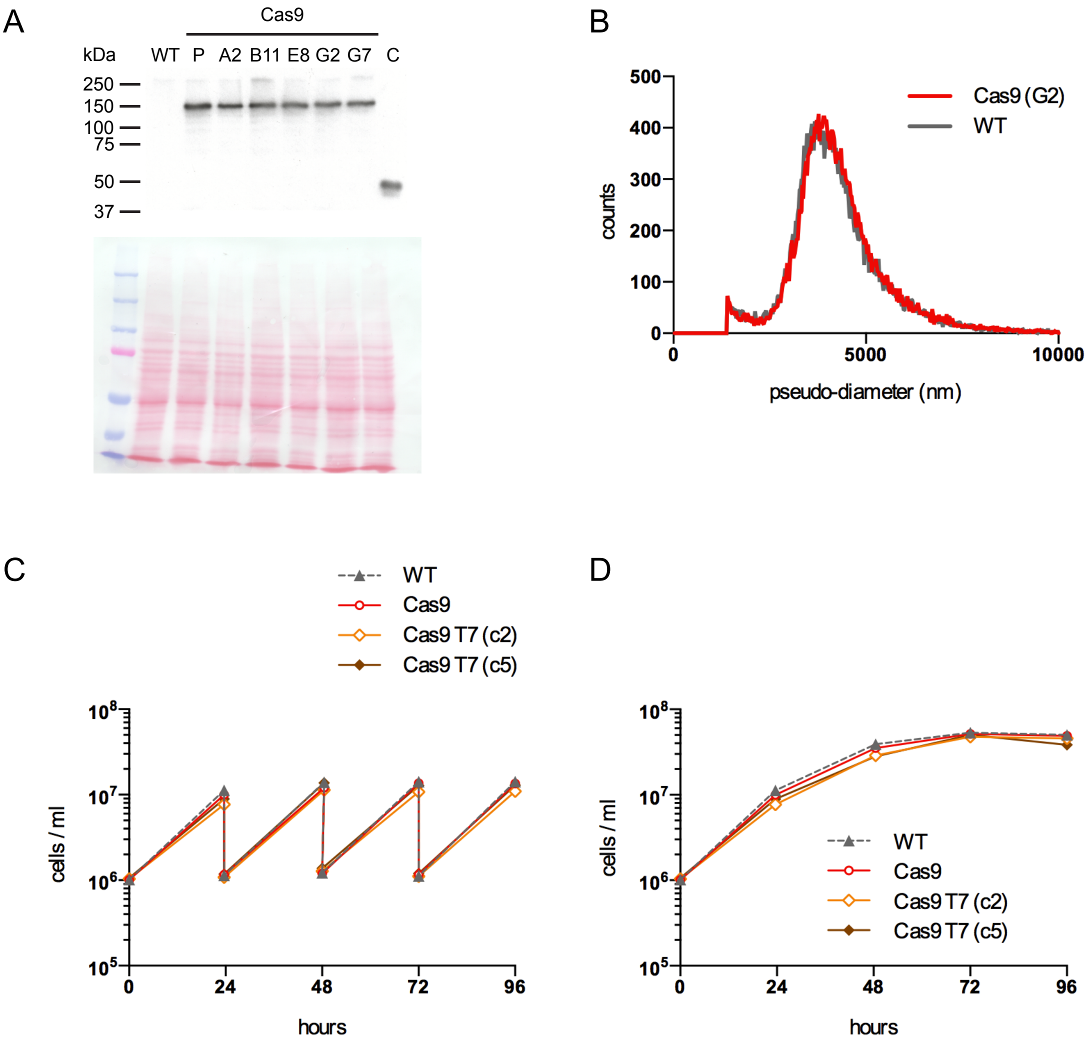

A
Cas9
kDa
WT
P
A2
B11
E8
G2
G7
C
250
150
100
75
50
37
B
Cas9 (G2)
WT
counts
pseudo-diameter (nm)
C
WT
Cas9
Cas9 T7 (c2)
Cas9 T7 (c5)
cells / ml
hours
D
WT
Cas9
Cas9 T7 (c2)
Cas9 T7 (c5)
cells / ml
hours

Fig. S1

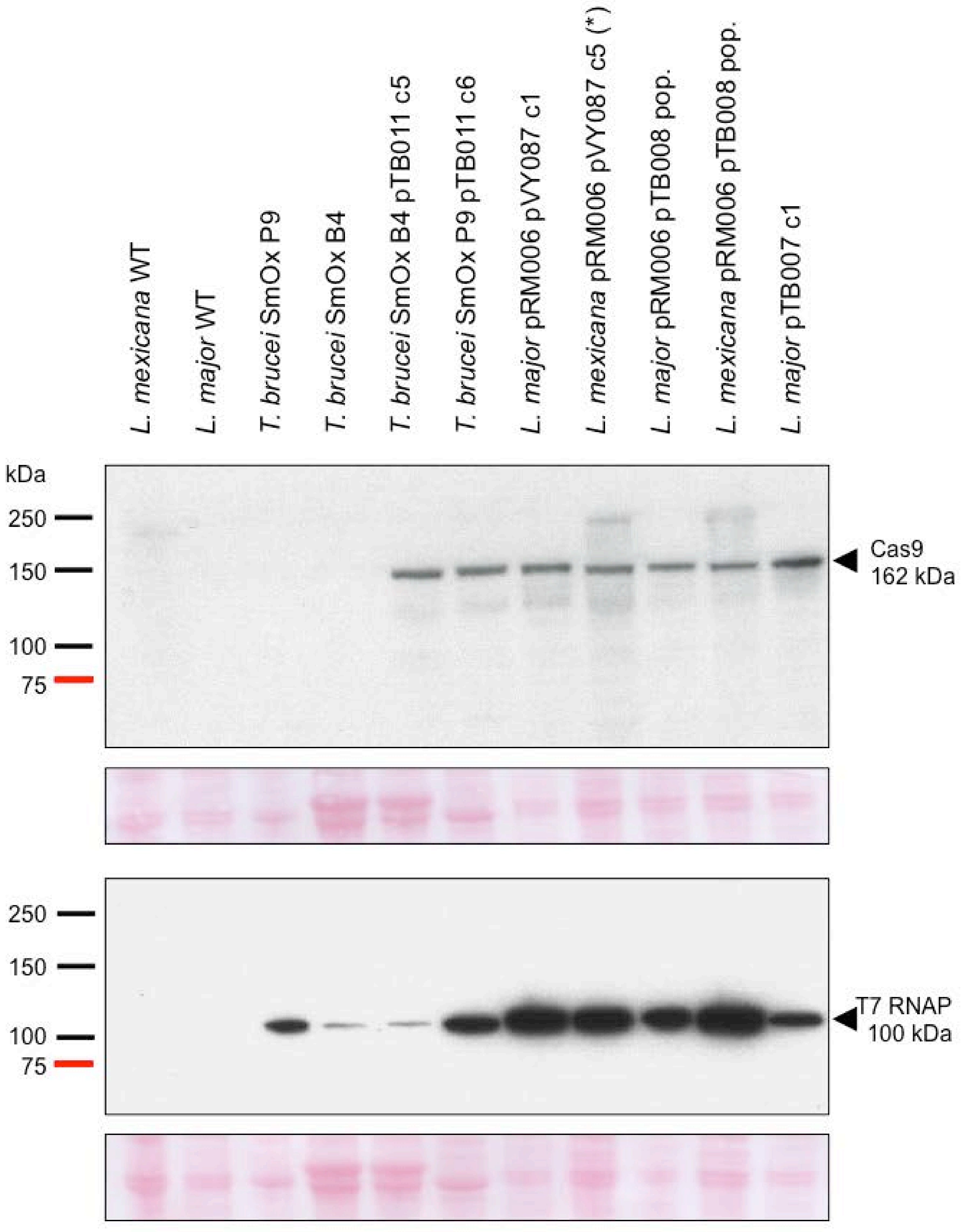
L. mexicana WT
L. major WT
T. brucei SmOx P9
T. brucei SmOx B4
T. brucei SmOx B4 pTB011 c5
T. brucei SmOx P9 pTB011 c6
L. major pRM006 pVY087 c1
L. mexicana pRM006 pVY087 c5 (*)
L. major pRM006 pTB008 pop.
L. mexicana pRM006 pTB008 pop.
L. major pTB007 c1
kDa
250
150
100
75
Cas9
162 kDa
250
150
100
75
T7 RNAP
100 kDa

Fig. S2

pPLOT Blast-mNeonGreen-Blast

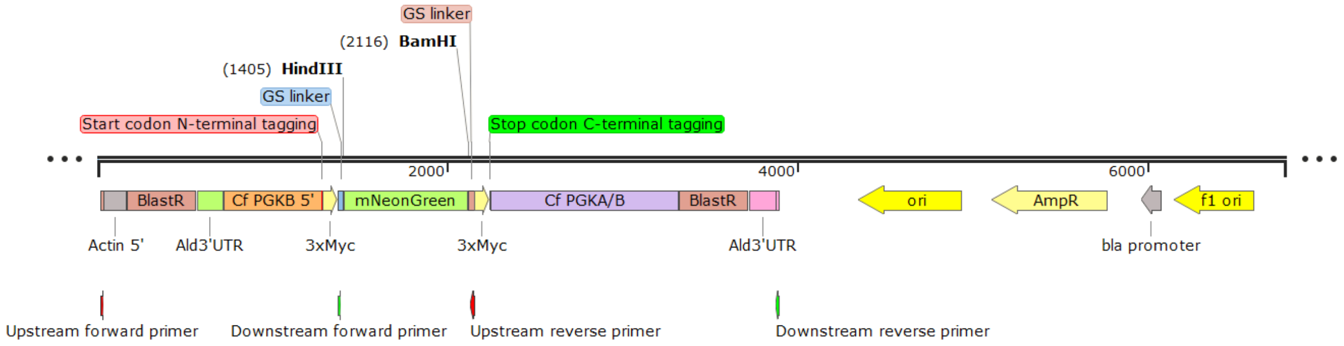

pTBlast

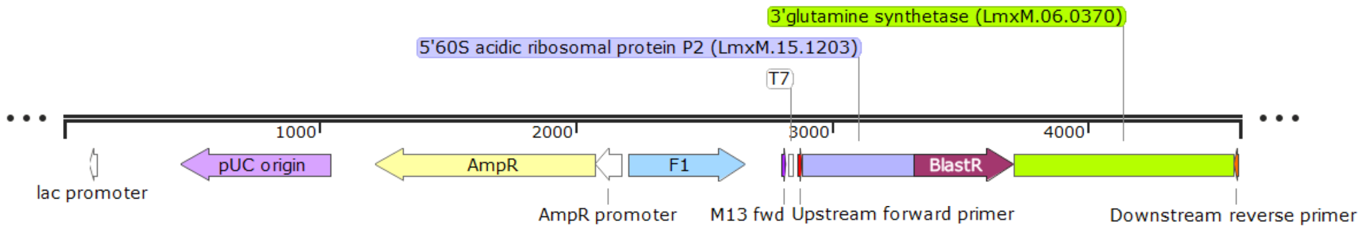

pTPuro

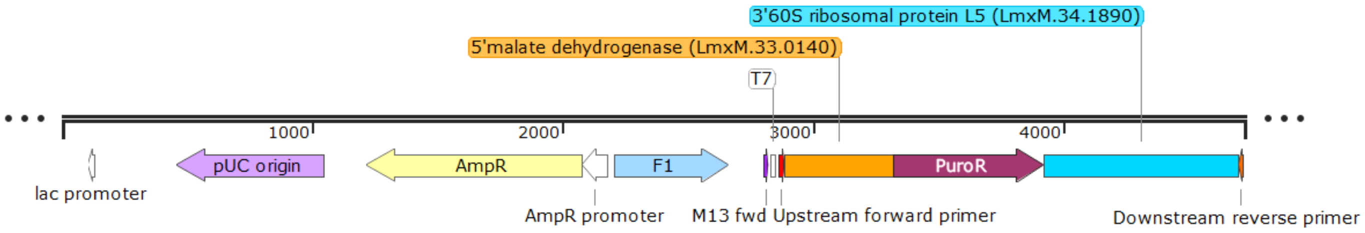

pTNeo

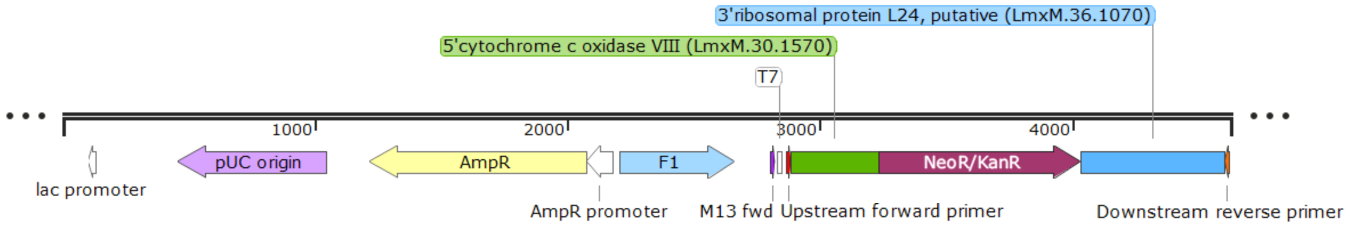

Fig. S3

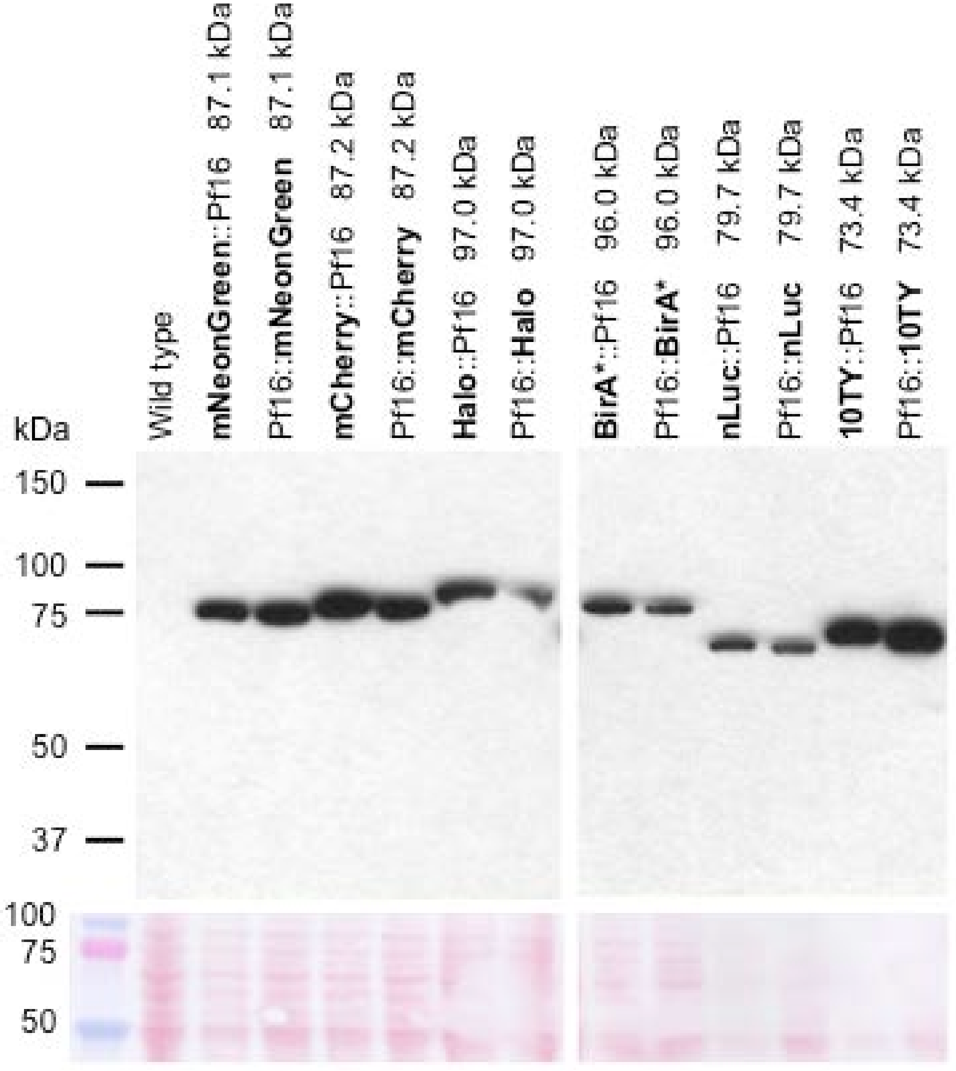
kDa
150
100
75
50
37
100
75
50
Wild type
mNeonGreen::Pf16 87.1 kDa
Pf16::mNeonGreen 87.1 kDa
mCherry::Pf16 87.2 kDa
Pf16::mCherry 87.2 kDa
Halo::Pf16 97.0 kDa
Pf16::Halo 97.0 kDa
BirA*::Pf16 96.0 kDa
Pf16::BirA* 96.0 kDa
nLuc::Pf16 79.7 kDa
Pf16::nLuc 79.7 kDa
10TY::Pf16 73.4 kDa
Pf16::10TY 73.4 kDa

Fig. S4

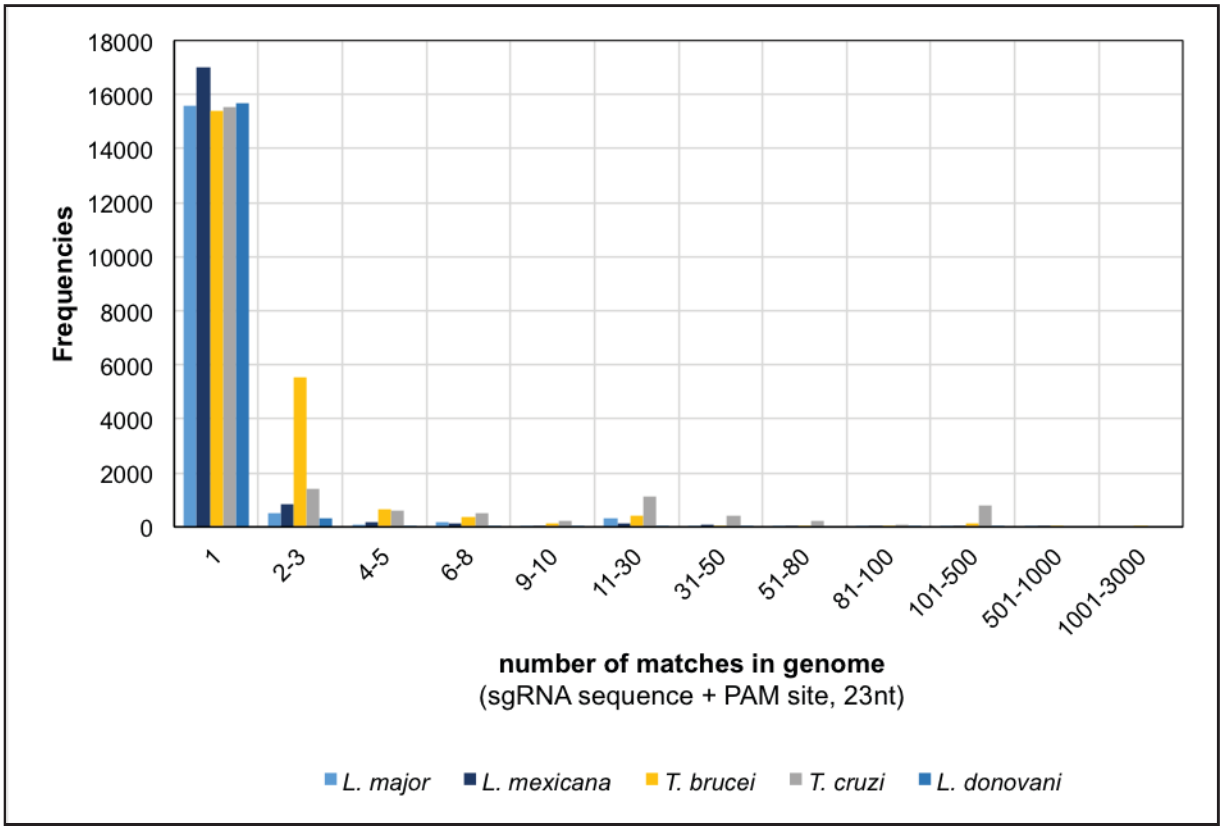

18000
16000
14000
12000
10000
8000
6000
4000
2000
0
Frequencies
1
2-3
4-5
6-8
9-10
11-30
31-50
51-80
81-100
101-500
501-1000
1001-3000
number of matches in genome
(sgRNA sequence + PAM site, 23nt)
L. major
L. mexicana
T. brucei
T. cruzi
L. donovani

Fig. S5

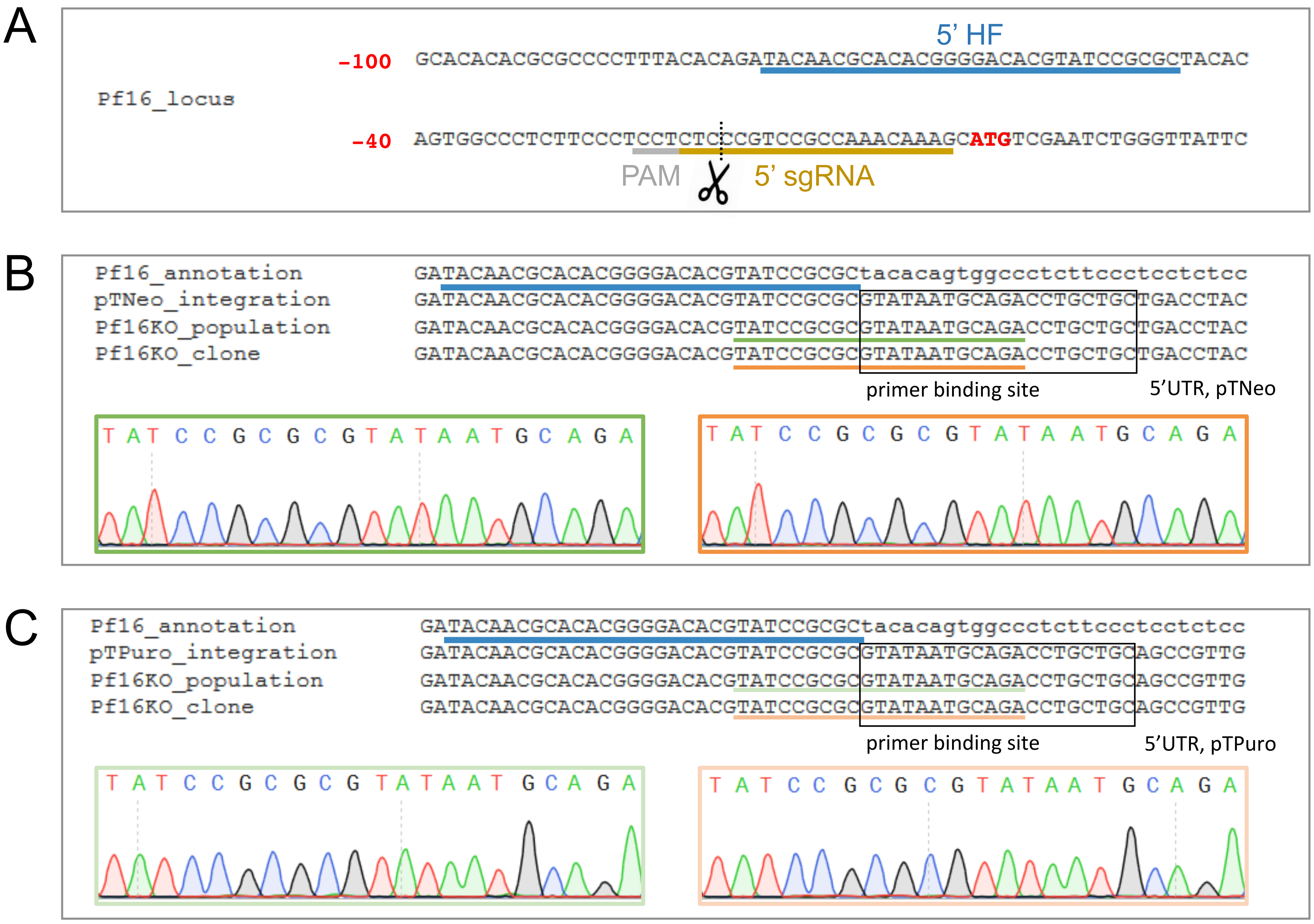
A
5' HF
-100 GCACACACGCGCCCCTTTACACAGATACAACGCACACGGGGACACGTATCCGCGCTACAC
Pf16_locus
-40 AGTGGCCCTCTTCCCTCCTCTCCCGTCCGCCAAACAAAGCATGTCGAATCTGGGTTATTC
PAM
5' sgRNA
B
Pf16_annotation GATACAACGCACACGGGGACACGTATCCGCGCtacacagtggccctcttccctcctctcc
pTNeo_integration GATACAACGCACACGGGGACACGTATCCGCGCGTATAATGCAGACCTGCTGCTGACCTAC
Pf16KO_population GATACAACGCACACGGGGACACGTATCCGCGCGTATAATGCAGACCTGCTGCTGACCTAC
Pf16KO_clone GATACAACGCACACGGGGACACGTATCCGCGCGTATAATGCAGACCTGCTGCTGACCTAC
primer binding site
5'UTR, pTNeo
C
Pf16_annotation GATACAACGCACACGGGGACACGTATCCGCGCtacacagtggccctcttccctcctctcc
pTPuro_integration GATACAACGCACACGGGGACACGTATCCGCGCGTATAATGCAGACCTGCTGCAGCCGTTG
Pf16KO_population GATACAACGCACACGGGGACACGTATCCGCGCGTATAATGCAGACCTGCTGCAGCCGTTG
Pf16KO_clone GATACAACGCACACGGGGACACGTATCCGCGCGTATAATGCAGACCTGCTGCAGCCGTTG
primer binding site
5'UTR, pTPuro

Fig. S6

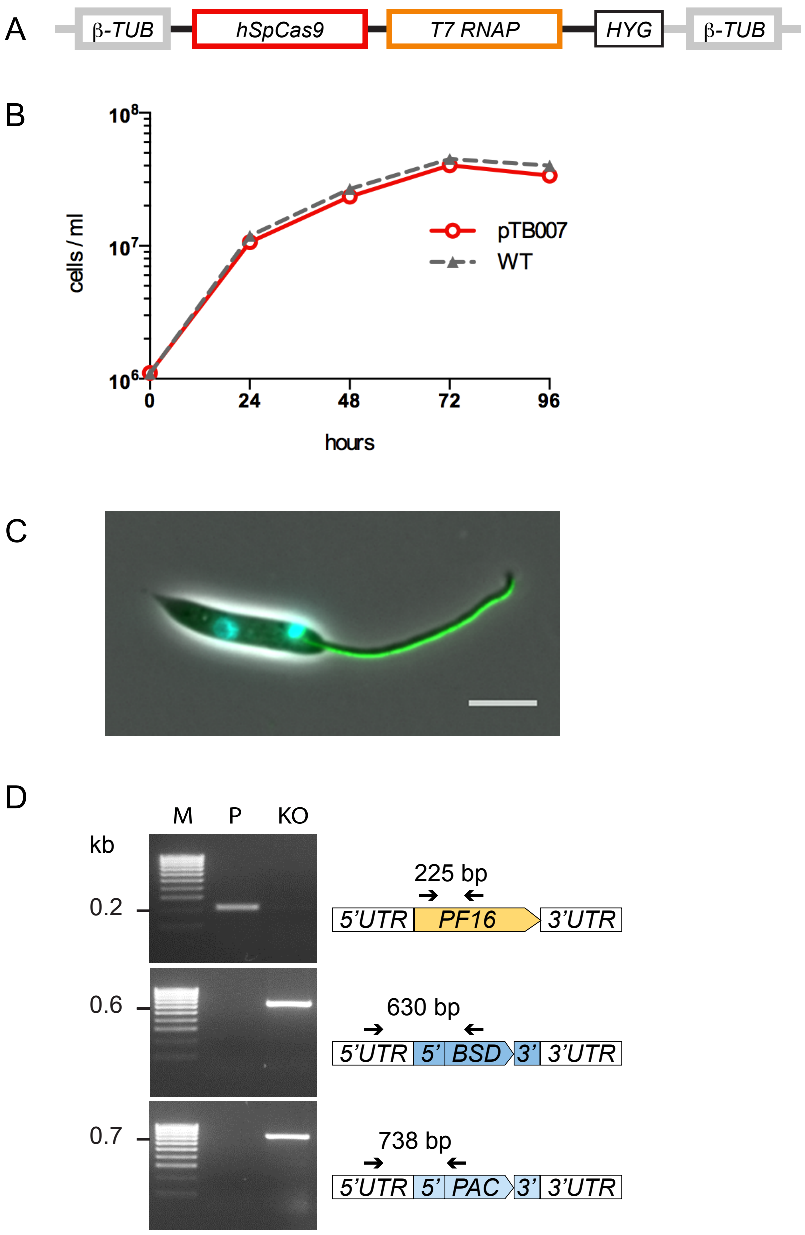
A
β-TUB
hSpCas9
T7 RNAP
HYG
β-TUB
B
10^8
10^7
10^6
cells / ml
0
24
48
72
96
hours
pTB007
WT
C
D
M
P
KO
kb
0.2
0.6
0.7
225 bp
5'UTR
PF16
3'UTR
630 bp
5'UTR
5'
BSD
3'
3'UTR
738 bp
5'UTR
5'
PAC
3'
3'UTR

Fig. S7

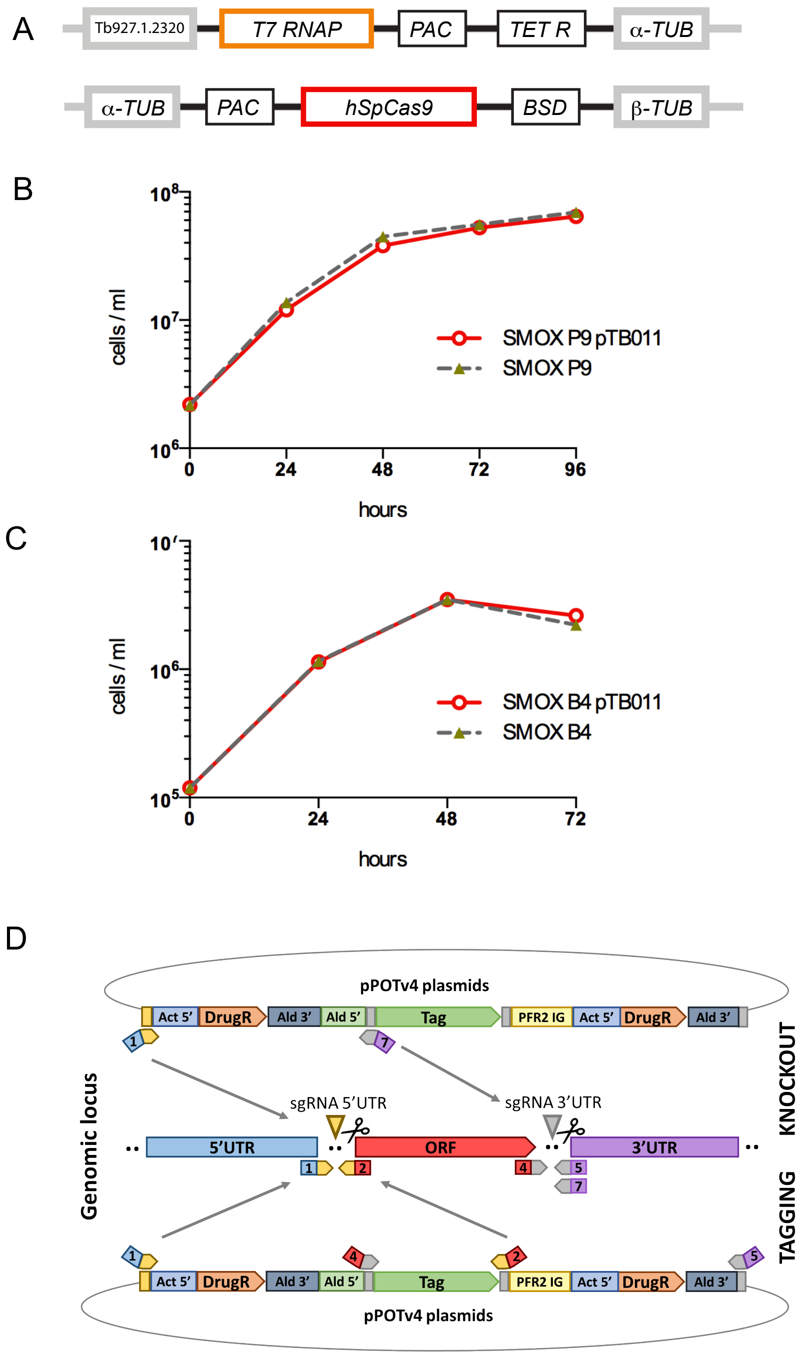

A
Tb927.1.2320
T7 RNAP
PAC
TET R
α-TUB
α-TUB
PAC
hSpCas9
BSD
β-TUB
B
cells / ml
hours
SMOX P9 pTB011
SMOX P9
C
cells / ml
hours
SMOX B4 pTB011
SMOX B4
D
pPOTv4 plasmids
Act 5'
DrugR
Ald 3'
Ald 5'
Tag
PFR2 IG
KNOCKOUT
Genomic locus
sgRNA 5'UTR
sgRNA 3'UTR
5'UTR
ORF
3'UTR
TAGGING

Fig. S8

Figure Legends

Figure S1. Expression of Cas9 and T7 RNAP

(A) Upper panel: Whole cell lysates of *L. mexicana* wild type (WT), the uncloned *L. mex* Cas9 population (P) and five clonal *L. mex* Cas9 cell lines (A2, B11, E8, G2 and G7) were analysed in a Western blot probed with anti-FLAG. The calculated MW for hSpCas9 is 165kDa. C, positive control containing a 41 kDa protein with a FLAG epitope (Abcam, ab5395). Lower panel: the membrane stained with Ponceau S before antibody detection. (B) histogram showing cell volumes of *L. mexicana* wild type cells and *L. mex* Cas9 clone G2 measured using a CASY cell counter. (C, D) Growth curves of *L. mexicana* wild type, *L. mex* Cas9 clone G2, *L. mex* Cas9 T7 clones c2 and c5. Cultures were seeded at $1x10^6$ cells/ml and cells were counted and diluted back to $1x10^6$ cells/ml every 24 h (C) or left to grow to stationary phase (D).

Figure S2. Detection of Cas9 and T7 RNAP in Western blots of *Leishmania* spp. and *T. brucei* cell lines

Western blot of whole cell lysates probed with anti-Flag (upper-panel) or anti-T7 RNAP (lower-panel). The asterisk (*) indicates the *L. mex* Cas9 T7 cell line. The Ponceau S-stained membrane for each blot, showing the tubulin band, is included as a loading control.

Figure S3. Detailed maps of pPLOT and pT

Maps of pT and pPLOT plasmids. Dotted lines indicate site where they were linearized for display purposes. The 5' and 3' UTRs flanking the drug resistance genes in pTBlast, pTPuro and pTNeo are labelled with LmxM GeneIDs indicating the origin of the UTR sequences.

Figure S4. Detection of pPLOT tags on Western blot

The *L. mexicana PF16* gene was tagged in its endogenous locus with pPLOT amplicons encoding different tags; for each tag two cell lines were produced, to generate N- or C-

terminal fusions. Upper panel: Western blot of whole cell lysates tagged with an anti-myc antibody. Lower panel: the membrane stained with Ponceau S before antibody detection. Tagged nLuc runs slightly faster than its calculated MW.

Figure S5. sgRNA target counts for automated primer design

Each selected sgRNA target (including the PAM site) was aligned to the relevant genome and the number of matches was counted. The histogram shows frequencies for counted numbers of matches; the majority of sgRNAs match a unique site in the genome.

Figure S6. Sequence of edited *PF16* locus

(A) 5' end of the *PF16* gene sequence. The location of the 5'HF, 20 nt gRNA target and cleavage site are indicated. Red numbers indicate nucleotide positions relative to the start codon and red letters denote the start codon. PAM, protospacer adjacent motif. (B, C) Sequence alignment of the annotated *PF16* sequence and expected sequence resulting from integration of the pT drug resistance cassettes pTNeo (B) or pTPuro (C) and sequencing reads obtained from the Δ*PF16* population and a Δ*PF16* clone; green and orange boxes show the sequencing chromatograms.

Figure S7. Tagging and knockout of *PF16* in *L. major*

(A) Diagram showing *hSpCas9* and *T7 RNAP* integrated in the *β-tubulin* locus (*β-TUB)* of *L. major*. (B) Growth curve of *L. major* wild type (WT) and *L. major* pTB007. (C) Micrograph showing flagellar localisation of PF16::mNG in *L. major* pTB007 co-transfected with a tagging cassette amplified from pPLOT and *PF16* sgRNA template. Phase contrast image merged with mNG fluorescence (green) and Hoechst-stained DNA fluorescence (cyan). Scale bar 5 μm. (D) PCR analysis of the *L. major* pTB007 Δ*PF16* cell line. Left panel: PCR products visualised on agarose gel. M, 100 bp DNA ladder; P, parental cell line *L. major* pTB007; KO, Δ*PF16* population. Right panel: diagram showing the *PF16* locus and PCR primers (arrows)

used to test for presence of the CDS or correct integration of drug resistance genes (blue boxes).

Figure S8. ***T. brucei*** **cell lines expressing Cas9 and T7 RNAP**

(A) Diagram showing location of *T7 RNAP* upstream of the tubulin (*TUB*) array in *T. brucei* SmOx cell lines (adapted from Poon *et al*., 2012. *Open Biology*. **2**, 110037; *TET R* denotes Tetracyclin repressor protein gene) and pTB011 integrated in the tubulin locus for expression of hSpCas9. (B, C) Growth curves of procyclic form *T. brucei* SmOx P9, bloodstream form SmOx B4 and their pTB011-transfected derivatives. (D) Strategy for donor DNA amplification from pPOTv4 plasmids (Dean *et al*., 2015. *Open Biology*. **5**, 140197). primer pairs 1/2 and 4/5 amplify N- and C-terminal tagging cassettes, respectively; primer pair 1/7 amplifies knockout cassettes. Primers include 30 nt HF for double-strand break repair by homologous recombination. Primers 3 and 6 (not shown) are used to amplify the 5’ and 3’ sgRNA templates.

Supplementary methods

Antibodies and buffers used in Western blots

**Antibody**	**Blocking buffer**	**1st Ab dilution**	**Wash buffer**	**2n Ab buffer**
Anti-T7 RNAP, Novagen 70566	TBST* + 1% gelatin	1:2,500 in TBST	TBST	Anti-mouse 1:20,000 TBST + 1% milk (Marvel)
Anti-myc, Millipore 05-724	TBST + 5% milk (Marvel)	1:3,000 in TBST + 5% milk (Marvel)	TBST + 5% milk (Marvel)	Anti-mouse 1:20,000 TBST + 5% milk (Marvel)
Anti-FLAG M2, Sigma F3165	TBST + 5% milk (Marvel)	1:25,000 in TBST + 5% milk (Marvel)	TBST + 5% milk (Marvel)	Anti-mouse 1:20,000 TBST + 5% milk (Marvel)
Anti-LPG, LT22 [1]	TBST + 5% milk (Marvel)	1:1,000 in TBST + 5% milk (Marvel)	TBST	Anti-mouse 1:20,000 TBST + 5% milk (Marvel)
Anti-GPI-PLC [2]	TBST + 5% milk (Marvel)	1:1,500 in TBST + 5% milk (Marvel)	TBST + 5% milk (Marvel)	Anti-rabbit 1:5,000 in TBST + 5% milk (Marvel)

*TBST: Tris-buffered saline with 0.05% Tween 20

1. Ilg T, Harbecke D, Wiese M, Overath P (1993) Eur J Biochem 217: 603-615.

2. Sunter J, Webb H, Carrington M (2013) PLoS Pathog 9: e1003566.

PCR Primers

All sequences are written in the 5’ to 3’ orientation. Underlined sequences indicate sgRNA target sites.

sgRNA transcription

sgRNA forward (n, gene-sepcific target sequence)	GAAATTAATACGACTCACTATAGGnnnnnnnnnnnnnnnnnnnnGTTTTAGAGCTAGAAATAGC
G00	AAAAGCACCGACTCGGTGCCACTTTTTCAAGTTGATAACGGACTAGCCTTATTTTAACTTGCTATTTCTAGCTCTAAAAC

Recombination rate measurement

PF16 3’ sgRNA forward	GAAATTAATACGACTCACTATAGGTGCAGCAGCACTAGCGGGGGAGGGTTTTAGAGCTAGAAATAGC

mCherry / eYFP double tagging of PF16

PF16 eYFP forward	333: AAGATCGAGAACTACCACGTGCAGCAGCACGTGAGCAAGGGCGAGGAGCTGTT
PF16 eYFP reverse	334: GCAGCGTGCATGGGCGTGACTGTGCCGAAATTAGCCCTCCCACACATAACCAGAG
PF16 mCherry forward	335: AAGATCGAGAACTACCACGTGCAGCAGCACACTAGTATGGTGAGCAAGGGCGAGG
PF16 mCherry reverse	336: GCAGCGTGCATGGGCGTGACTGTGCCGAAATTACACTTTATGCTTCCGGCTCGTATGTTG
PF16 sgRNA forward	295:<br>GAAATTAATACGACTCACTATAGGCGGATGCTCAGCGGGCCTTTGTTTTAGAGCTAGAAATAGC

Construction of pT plasmids

	5’UTR, 1st reaction	3’UTR, 1st reaction	Nested primers, 2nd second reaction
pTBlast	478F:<br>TTAGCAATCTGAGCAATCTGCCGGATGAAAACGAGAGAGAGAC	480F:<br>CTCTGGTTATGTGTGGGAGGGCTAAATAGAAGCCACACCGCGTGAG	482F:<br>GTCGCAATTTAAATGTATAATGCAGACCTGCTGCCGGATGAAAACGAGAGAGAGAC
	479R:<br>GGGTGGATTCTTCTTGAGACAAAGGCATGGTGGAGCGAGGATACGAG	481R:<br>TCTTAGTACGAACTGCAATGCAACCAGTGTGTCAGTTGGAGAGA	483R:<br>CGAGTCATTTAAATCCAATTTGAGAGACCTGTGCAACCAGTGTGTCAGTTGGAGAGA
pTNeo	502F:<br>TTAGCAATCTGAGCAATCTGCTGACCTACGCAGCCTTTTGCG	504F:<br>CTATCGCCTTCTTGACGAGTTCTTCTGAAGCTCCGCCGGTGTTCGTC	506F:<br>GTCGCAATTTAAATGTATAATGCAGACCTGCTGCTGACCTACGCAGCCTTTTGCG
	503R:<br>TCCATCTTGTTCAATGGCCGATCCCATAGCCTTCGAGTATGAGCTGCTTT	505R:<br>TCTTAGTACGAACTGCAATGCAGATGAGAAAGCGCGGCCATC	507R:<br>CGAGTCATTTAAATCCAATTTGAGAGACCTGTGCAGATGAGAAAGCGC

			GGCCATC
pTPuro	496F: TTAGCAATCTGAGCAATCTGCAGCCGTTGCATTGGTGGTCGT	498F: CATGACACGTAAGCCGGGAGCCTAAGTCGAGCGCTCACCTCTGAA	500F: GTCGCAATTTAAATGTATAATGCAGACCTGCTGCAGCCGTTGCATTGGTGGTCGT
	497R: GACGAACCGTTGGCTTGTATTCAGTCATTATGAAAGACCGCAAAAGAAAATGTG	499R: TCTTAGTACGAACTGCAATGCGTCGTTGTAGTTCATGGAGACAC	501R: CGAGTCATTTAAATCCAATTTGAGAGACCTGTGCGTCGTTGTAGTTCATGGAGACAC

Construction of pPLOT plasmids

5’PGKB myc-tag forward	473: TTAGCAAAGCTTAGAACCGGAACCGGAACCACTACCAGAACCCAGGTCCTCCTCGCTAATCAGCTTCTGCTCGAGGTCCTCCTCGCTAATGA
5’PGKB myc-tag reverse	474: AACTATCTTCCACTTGTCAAGCATGGAACAGAAGCTGATCAGCGAAGAAGACCTGGAGCAAAAGCTCATTAGCGAGGAGGACCTCGAGCA
5’PGKB second reaction	472: TTAGCAACGCGTTCGAGACCGACAAGACCAGAAT
PGKB/A myc-tag forward	475: TTAGCAGGATCCGGATCAGGATCTGGATCAGGATCGGGTAGTGAACAGAAGCTGATCAGCGAAGAAGACCTGGAGCAAAAGCT
PGKB/A myc-tag reverse	476: GAATTCTCAGTGGTGGTGGTGGTTACAGGTCCTCCTCGCTAATCAGCTTCTGCTCGAGGTCCTCCTCGCTAATGAGCTTTTGCTCCAGGTCTTCTTCGC
PGKB/A second reaction	477: TTAGCAGCTAGCCGCTTGACAAGTGGAAGATAGTTG

Construction of addback plasmids

PF16 forward	533ABF: GCAATCACTAGTCAAACAAAGCATGTCGAATCGGG
PF16 reverse	533ABR: GTCGCAGAATTCCCTCCTCCCCCGCTAGTGCTGCT
LPG1 forward	LPG_350: TTACGTACTAGTCACAGAGAAATGGCGCCG
LPG1 reverse	LPG_351: TTACGTCCTCGAGBAGAATGCTTAACGGGAGCGA

Tagging and knockout of PF16 and LPG1 with pT / pPLOT plasmids in *L. mexicana*

Lmex PF16 5’HF forward	533NF: TACAACGCACACGGGGACACGTATCCGCGCgtataatgcagacctgctgc
Lmex PF16 5’HF reverse	533NR: GAAGGTTTGCAGAATAACCCGATTCGACATactacccgatcctgatccag
Lmex PF16 3’HF forward	533CF: AAGATCGAGAACTACCACGTGCAGCAGCACggttctggtagtggttccgg
Lmex PF16 3’HF reverse	533CR: CGCGACGAGCAGCGTGCATGGGCGTGACTGccaatttgagagacctgtgc
Lmex PF16 5’ sgRNA forward	GAAATTAATACGACTCACTATAGGCTTTGTTTGGCGGACGGGAGGTTTTAGAGCTAGAAATAGC
Lmex PF16 3’	GAAATTAATACGACTCACTATAGGCGGATGCTCAGCGGGCCTTTGTTTTAGAGCTAGAAATAGC

sgRNA forward	
LPG1 5'HF forward	LPG_313: GAGGACGCGCGCCGGTTATCGTTACCTAGAgtataatgcagacctgctgc
LPG1 3'HF reverse	LPG_315: CCAACTTGCGGAATGCGCGCGTCGCAACGGccaatttgagagacctgtgc
LPG1 5' sgRNA forward	GAAATTAATACGACTCACTATAGGTGTTTCTACGAGGAGGCACTGGGGTTTTAGAGCTAGAAATAGC
LPG1 3' sgRNA forward	GAAATTAATACGACTCACTATAGGGGGAGCGTGTGCGGCGCATCAGGGTTTTAGAGCTAGAAATAGC

Tagging and knockout of PF16 with pT / pPLOT plasmids in *L. major*

LmjF PF16 5'HF forward	587NF: CGTATCAGCGCTACACAGTGGCCCTCTCCTgtataatgcagacctgctgc
LmjF PF16 5'HF reverse	587NR: GAAGGTTTGCAGAATAACCCGATTCGACATactacccgatcctgatccag
LmjF PF16 3'HF forward	587CF: AAGATCGAGAACTACCACGTGCAGCAGCACggttctggtagtggttccgg
LmjF PF16 3'HF reverse	587CR: CGAGCAGCGTGCGTGGGCGTGACTATGCCGccaatttgagagacctgtgc
LmjF PF16 5' sgRNA forward	GAAATTAATACGACTCACTATAGGCTTTGTGTGGCGGCGAGGAGGTTTTAGAGCTAGAAATAGC
LmjF PF16 3' sgRNA forward	GAAATTAATACGACTCACTATAGGGCTGATGCTCAGCCGCCATTGTTTTAGAGCTAGAAATAGC

Tagging and knockout of GPI-PLC

GPI-PLC 5'HF forward	593NF: AAAGAAAGAAAGAAAGAAAGAAAGAAAAAGGTATAATGCAGACCTGCTGC
GPI-PLC PF16 5'HF reverse	593NR: CTGCGGTGACCACTTTACACCACCAAACATACTACCCGATCCTGATCCAG
GPI-PLC PF16 3'HF forward	593CF: CATTTAAACACCAACCAAACCGCAAGGTCAGGTTCTGGTAGTGGTTCCGG
GPI-PLC PF16 3'HF reverse for tagging	593CR: CACTTAACTTTTTTTTTACTTTTAAACCCTCCAATTTGAGAGACCTGTGC
GPI-PLC PF16 3'HF reverse for knockout	593CRF: CACTTAACTTTTTTTTTACTTTTAAACCCTCCGGAACCACTACCAGAACC
GPI-PLC PF16 5' sgRNA forward	GAAATTAATACGACTCACTATAGGAGGGGAAGGGGACTTTGAGAGTTTTAGAGCTAGAAATAGC
GPI-PLC PF16 3' sgRNA forward	GAAATTAATACGACTCACTATAGGCTGCGTTTGTGACTGAAACAGTTTTAGAGCTAGAAATAGC

Knockout validation

ORF amplification	*Lmex* PF16 ORF forward	533OF: CCTTCGACGAGTACCAGAAGGCA

	Lmex PF16 ORF reverse	533OR: CATCTCCAGACACGACGTTCTCG
	LmajF PF16 ORF forward	587OF: GACGAGTACCAGAAGGCACG
	LmajF PF16 ORF reverse	587OR: CATCTCCAGAAACGACGTTC
	LPG1 ORF forward	LPG_334: ATTAGTGCGTATCGGCCTGT
	LPG1 ORF reverse	LPG_335: AATGACAGCGAATATTCTCGC
	GPI-PLC forward	593OF: TCATGGATGAGTGACACGCG
	GPI-PLC reverse	593OR: AAAGATTTTGACAACGTCCC
Amplification of sequence across integration junction, *Lmex* PF16	*Lmex* PF16 5'UTR forward	533UF: GACGTCGCTGCAGAAGTTATCCT
	Neomycin reverse	519: GTCTTGACAAAAAGAACCGGGC
	Puromycin reverse	558: TCAATGTGTCGATCTGGGTCAAC
Amplification of sequence across integration junction, LPG1	LPG1 3'UTR reverse	LPG_331: TTGATTCGGTGATGGCTCG
	Blasticidin forward	LPG_127: CTGCATCCTGGGATCAAAGC
	Neomycin forward	LPG_319: ACCGCTTCCTCGTGCTTTA
Amplification of sequence across integration junction, *LmajF* PF16	*LmajF* PF16 5'UTR forward	587UF: TGACATTGCTGCCGAAGTGA
	Blastacidin reverse	518A: CCGTTGCTCTTTCAATGAGGGTG
	Puromycin reverse	558: TCAATGTGTCGATCTGGGTCAAC
Amplification of sequence across integration junction, GPI-PLC	GPI-PLC 5'UTR forward	593UF: GTCGTCGTAGTTGTTGTTAT
	Neomycin reverse	519: GTCTTGACAAAAAGAACCGGGC
	Hygromycin reverse	JV78: CAGCTATTTACCCGCAGGAC
Sequencing primers for analysis of *Lmex PF16* knockout cell line	Neomycin integration	532: CTGTTCCTGTCGTACGAAAG
	Puromycin integration	533UF: GACGTCGCTGCAGAAGTTATCCT

Detection of sgRNA template

G00F	562: TTAATACGACTCACTATAGG
G00R	563: GCACCGACTCGGTGCCACTT
